# Genome sequence analysis of deep sea *Aspergillus sydowii* BOBA1 and effect of high pressure on biodegradation of spent engine oil

**DOI:** 10.1038/s41598-021-88525-9

**Published:** 2021-04-30

**Authors:** A. Ganesh Kumar, D. Manisha, K. Sujitha, D. Magesh Peter, R. Kirubagaran, G. Dharani

**Affiliations:** Marine Biotechnology Division, National Institute of Ocean Technology, Ministry of Earth Sciences (MoES), Government of India, Chennai, 600100 Tamil Nadu India

**Keywords:** Environmental biotechnology, Environmental sciences, Environmental microbiology

## Abstract

A deep-sea fungus *Aspergillus sydowii* BOBA1 isolated from marine sediment at a depth of 3000 m was capable of degrading spent engine (SE) oil. The response of immobilized fungi towards degradation at elevated pressure was studied in customized high pressure reactors without any deviation in simulating in situ deep-sea conditions. The growth rate of *A. sydowii* BOBA1 in 0.1 MPa was significantly different from the growth at 10 MPa pressure. The degradation percentage reached 71.2 and 82.5% at atmospheric and high pressure conditions, respectively, within a retention period of 21 days. The complete genome sequence of BOBA1 consists of 38,795,664 bp in size, comprises 2582 scaffolds with predicted total coding genes of 18,932. A total of 16,247 genes were assigned with known functions and many families found to have a potential role in PAHs and xenobiotic compound metabolism. Functional genes controlling the pathways of hydrocarbon and xenobiotics compound degrading enzymes such as dioxygenase, decarboxylase, hydrolase, reductase and peroxidase were identified. The spectroscopic and genomic analysis revealed the presence of combined catechol, gentisate and phthalic acid degradation pathway. These results of degradation and genomic studies evidenced that this deep-sea fungus could be employed to develop an eco-friendly mycoremediation technology to combat the oil polluted marine environment. This study expands our knowledge on piezophilic fungi and offer insight into possibilities about the fate of SE oil in deep-sea.

## Introduction

Anthropogenic oil spill contaminations in marine environment are frequent, posing a serious environmental problem and hence it is important to find an eco-friendly technology for bioremediation. The spilled oil on the sea water surface partially dissolves and diffuses in the water column reaching the sea bed^1^. Oil spills seriously affect the marine environment and it is vital to remediate these contaminants. In marine environment, biodegradation of oil depends on various parameters that include contents of the oil, availability of electron acceptors, temperature, pressure, and the dominant microbial metabolisms. Recent studies on bioremediation by fungi (mycoremediation) considered this tool as an efficient technology for treating a wide variety of pollutants^2,3^. Mycoremediation have multiple advantages, since fungi have the capability to penetrate hard contaminated sites and possess versatile enzymatic machinery for the degradation of toxic compounds^4^. In marine environment, fungi play an important role in biogeochemical cycles with their ability in removing and decomposing various compounds^5^. Fungal isolates with an ability to degrade polycyclic aromatic hydrocarbons (PAHs) are isolated from marine niches such as deep-sea waters^6^, sediments and tarballs^7^, oil soaked sand patties^8^ and marine sponges^9^. Oil spill contaminated sites are most preferred niches for hydrocarbon degrading microbes, interestingly, abundant hydrocarbon degraders were also observed in totally different environment like Mariana Trench at a depth of 10,400 m below the surface of the water^10^. The physical, chemical and biological interactions in ocean are very complex, and due to prevailing harsh environmental conditions, appropriate bioremediation technology is needed to treat the oil spill in surface and deep-sea environment. Microbial responses to the deep water oil spills^11^ and to high pressure conditions^12^ have triggered the idea of using deep-sea microorganisms in biodegradation. In addition, degradation of xenobiotic compounds by native microorganisms offers better degradation rates^13^ and the indigenous microbe based technology may be vital for intensive treatment. The presence of oil degrading fungi from marine environments has been investigated by various researchers. The marine fungi *Aspergillus sclerotiorum* and *Mucor racemosus* have been found to metabolize PAHs effectively^14^. Marine *Tolypocladium* sp. has been reported to degrade pyrene effectively about 95% after 7 days of incubation^15^. In addition, marine derived fungi *Cladosporium* sp., *Aspergillus sydowii, Pencillium citrinum, Mucor racemosus* were reported to degrade anthracene within 14 days^16^. Marine derived basidiomycetes *Marasmiellus* sp. has been found to degrade higher levels of pyrene and benzo[a]pyrene^9^. Some interesting findings have been made based on the potential functioning of marine fungi in biodegradation, bioremediation of PAHs and xenobiotic compounds^17,18^. However, only limited studies are available on the hydrocarbon biodegradation studies and growth of hydrocarbonoclastic piezotolerant fungi at high pressure conditions. The fungi growing under high hydrostatic pressure conditions may be potential candidate for developing new or improving existing bioremediation technology for restoration of polluted ecosystem. Under high hydrostatic pressure, several genes may be regulated and the interest on piezotolerant fungi is increasing due to their application in PAH degradation. In order to overcome the environmental problems caused by anthropogenic activities it is important to evaluate the potential of indigenous eukaryotic microorganisms from deep-sea sediment to target a particular pollutant. To achieve a successful mycoremediation, a new fungal species that possess catalytic enzymes and adequate mechanisms with high degradation potential must be selected. The use of fungi for bioremediation and degradation of recalcitrant organics in high pressure conditions has gained much interest in recent years, given their outstanding metabolic diversity and versatility. However, to our knowledge, there is no study to date about the ability of piezophilic fungi to degrade PAHs in high pressure conditions.

Research studies on cell growth and degradation of aromatic compounds identify that the ascomycetous fungi *Aspergillus* as a biocatalyst for environmental remediation. Several studies indicated that the genus *Aspergillus* is an interesting target to look for potential aromatic compounds degraders^19,20^. Immobilized microbial technology is favoured to enhance stress tolerance and to overcome the microbial loss in marine oil spill application. Thus in this study, we characterized the (1) biodegradation of SE oil by *A. sydowii* BOBA1 under ambient and elevated pressure conditions, (2) plausible biodegradation pathways of PAHs and (3) genes involved in PAHs degradation through Whole Genome Sequencing (WGS). This study is the first report on WGS of PAHs degrading *A. sydowii* BOBA1 and demonstrating the potential of mycoremediation under high pressure conditions.

## Results

### Isolation and identification of hydrocarbonoclastic deep-sea fungus

A strain BOBA1 isolated from 3000 m deep-sea sediment of the Bay of Bengal, India, was capable of degrading various PAHs and recalcitrant organic compounds. Analysis of substrate specificity confirmed that the BOBA1 grew well on substrates such as crude oil, SE oil, n-hexadecane, kerosene, petrol, brij-35, diesel, triton X-100, toluene, tween 80 and xylene. However, moderate growth was observed in silicone oil, sodium dodecyl sulphate, cedarwood oil and phenol, while no growth was observed in clove oil (Table 1). The sequence analysis of the ITS region exhibited 98% similarity with *Aspergillus sydowii*. The sequence of the ITS gene obtained was deposited in the NCBI genbank under accession number MN249529.

**Table 1:** Substrates specificity of *A. sydowii* BOBA1 indicates (˗) no growth, (++) moderate growth and (+++) luxuriant growth.

Substrate	Growth
Brij-35	+++
Cedar wood oil	++
Clove oil	˗
Crude oil	+++
Diesel	+++
n-Hexadecane	+++
Kerosene	+++
Petrol	+++
Phenol	++
Silicone oil	++
Sodium dodecyl sulphate	++
Spent engine oil	+++
Toluene	+++
Triton X-100	+++
Tween 80	+++
Xylene	+++

### Fungal growth physiology

The growth of *A. sydowii* BOBA1 at various culture conditions of pH, salinity and temperature was checked for possible bioremediation applications in challenging marine environment. The isolate grew on 10–40 °C with maximum growth at 25 °C. The growth was observed at various concentrations of NaCl ranging from 1 to 10% (w/v) with an optimum at 4% (w/v). This species was found to grow within a pH range of 4.0–10.0 with an optimum growth at pH 5. In MSM supplemented with different SE oil concentration (0.1, 1, 2, 5 and 10%), the fungal cells grew significantly up to 5% test concentration. The classification and general features of *A. sydowii* BOBA1 are described in Table 2.

**Table 2:** Classification and general features of *A. sydowii* BOBA1.

Property	Features
Classification	Domain: Fungi
	Phylum: Ascomycota
	Class: Eurotiomycetes
	Order: Eurotiales
	Family: Trichocomaceae
	Genus: *Aspergillus*
	Species: *sydowii*
	Strain: BOBA1
Cell shape	Conidial head with mycelium
Motility	Non-motile
Sporulation	Conidia
Optimum temperature for growth	25 °C
Optimum pH for growth	5
Optimum salinity (%)	4
Habitat	Marine habitat
Geographic location	Bay of Bengal
Sample collection	27 May 2017
Latitude	13º21.527′N
Longitude	80º53.077′E
Depth	3000 m

### Morphological characterization of immobilized fungus

**Figure 1:**
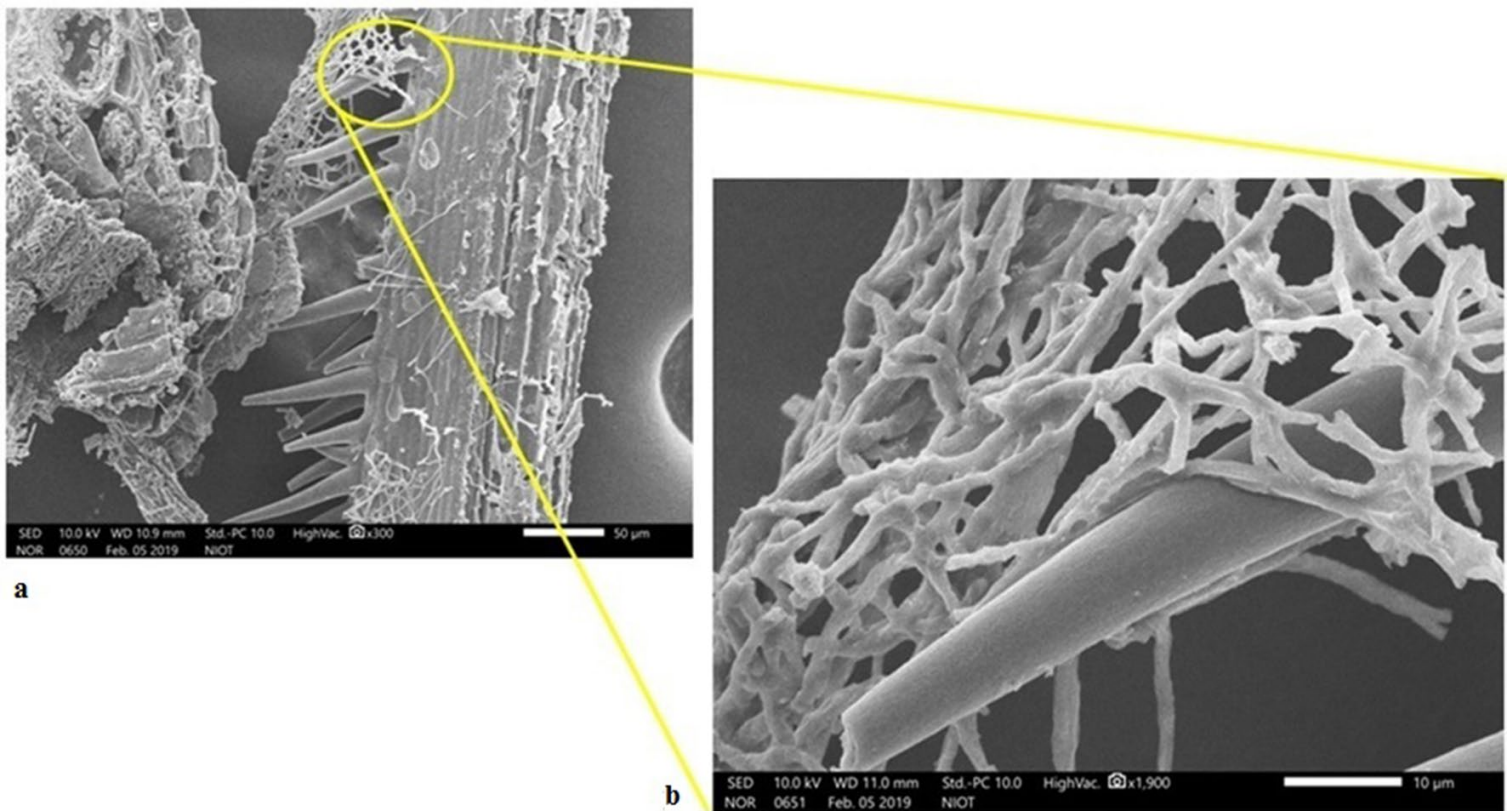
SEM image of immobilized *A. sydowii* BOBA1 (**a**). Dense mycelium with developed mass branching adhered to lignocellulose matrix (**b**). Enlarged image of the mycelial adherence on rice bran.

In this study, we investigated the immobilization of oil degrading fungus on rice bran (RB). *A. sydowii* BOBA1 cells were readily immobilized onto the RB surface, and the cell count of immobilized biomass reached 28.76 mg drycells/g supporting materials after incubation for 48 h. The microstructure of *A. sydowii* BOBA1 immobilized on RB was studied using SEM at higher magnification. RB consist of relatively intense and dense porous structure with a large internal surface area. Dense mycelium with well developed mass branching adhered to lignocellulose matrix was observed (Fig. 1).

### Effect of N and P fertilizers on degradation

To increase the efficacy of degradation by *A. sydowii* BOBA1, the effect of nitrogen (N) and phosphorus (P) fertilizers were performed. Addition of CO(NH_2_)_2_ as a nitrogen source increased the biomass concentration (60%) and oil degradation (72%). Moreover, a significant increment in oil degradation (54%) and biomass production (51%) was observed with the supplementation of (NH_4_)H_2_PO_4_.

### Degradation analysis: studies at ambient and high pressure conditions

**Figure 2:**
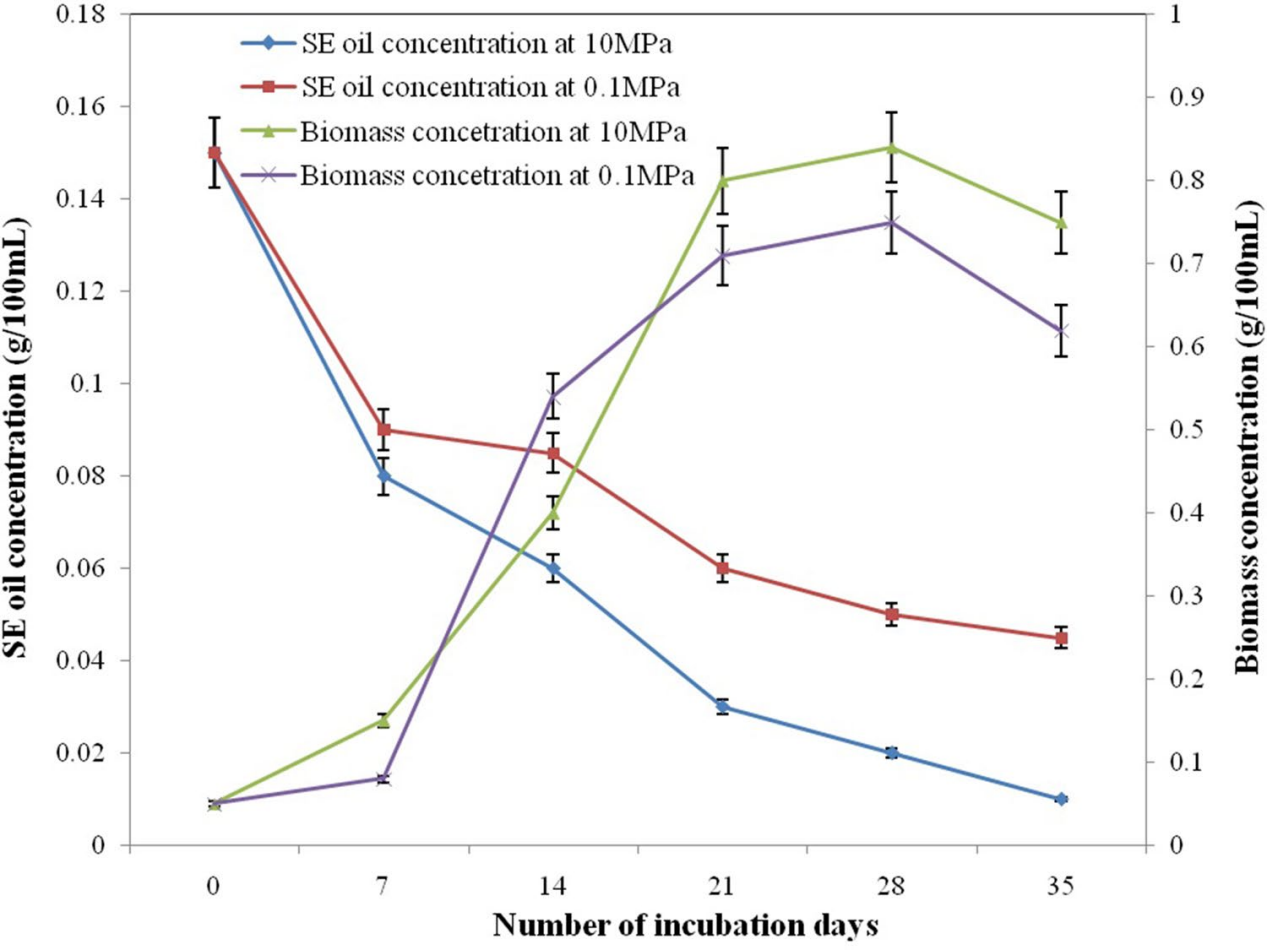
*A. sydowii* BOBA1 at different incubation time under 0.1 MPa and 10 MPa culture pressure conditions.

Biodegradation of SE oil 1 g/L by the immobilized *A. sydowii* BOBA1 was studied at every 7 days interval till 28 days under conditions of 0.1 MPa and 10 MPa pressure. At both conditions an initial lag phase around 0–7 days were required for the fungal cells to adapt at higher concentration of SE oil containing high concentrations of PAHs and to exhibit oil degradation efficiency. In 0.1 MPa pressure conditions, the oil degradation reached 71.2% at 21 days of incubation. In 10 MPa pressure condition, the cell biomass increased from 0.15 to 0.41 g/100 mL and oil degradation reached 82.5% within 21 days of incubation (Fig. 2). Interestingly, at high pressure conditions the SE oil in the MSM medium was considerably degraded. This result confirmed the piezotolerance and hydrocarbonoclastic potential of deep-sea *A. sydowii* BOBA1. Biological oxygen demand (BOD_5_) of control, 0.1 MPa and 10 MPa biologically treated samples were found to be 288 mg/L, 85 mg/L and 59 mg/L, respectively.

### Piezotolerance—SEM analysis

**Figure 3:**
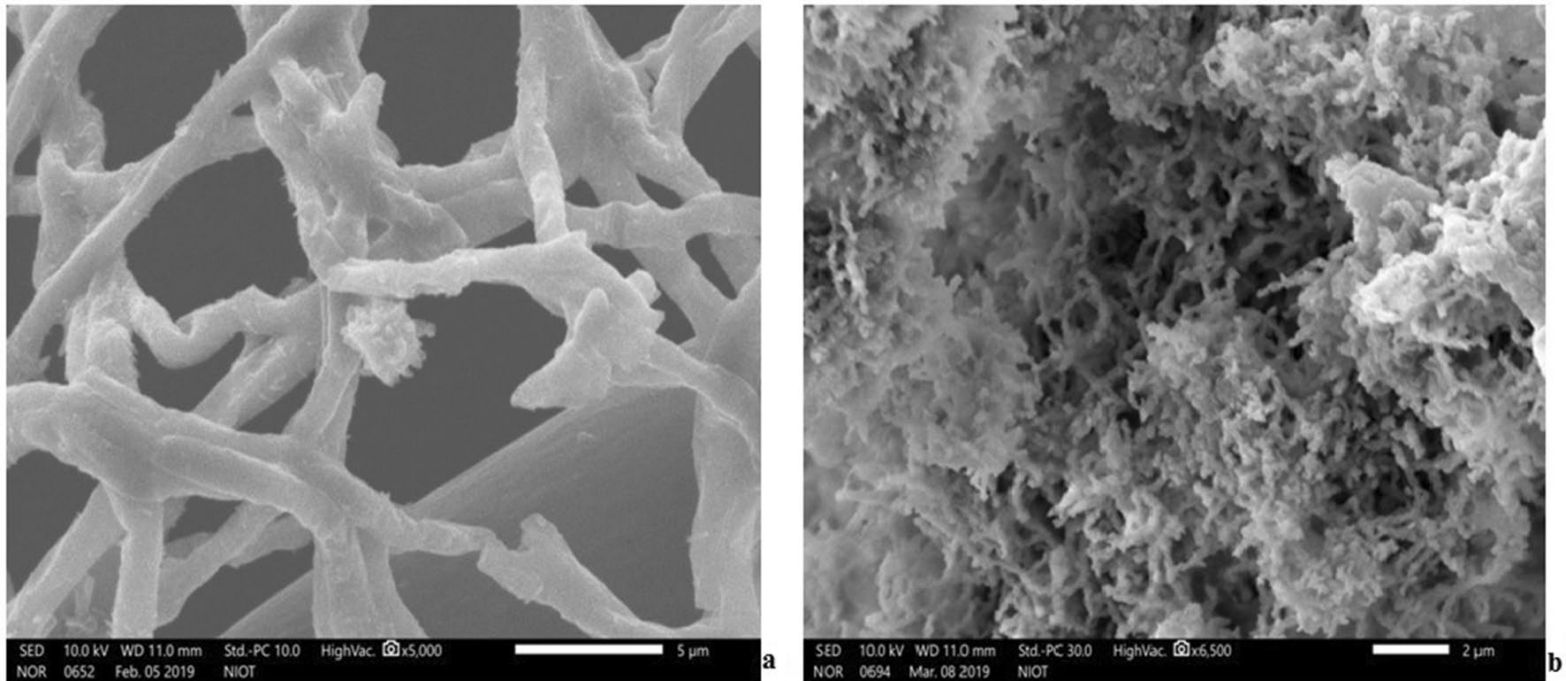
*A. sydowii* BOBA1 observed during degradation of SE oil by SEM. (**a**) Cells at 0.1 MPa. (**b**) Cell clusters at 10 MPa.

Deep-sea *A. sydowii* BOBA1 grew similarly and exhibited similar SE oil degradation at 0.1 and 10 MPa culture conditions. Figure 3 illustrates the changes in mycelium morphology observed at SEM analysis. In 0.1 MPa, fungi grow in dispersed form consisting of network like microstructure with mean hypha filament diameter of 1.3–1.8 µm. At 10 MPa pressure conditions, cell growth was more dense and reduced in size with an average hypha filament diameter of 0.1–0.13 µm (Fig. 3). The increased surface area at 10 MPa could enhance the metabolic activity of fungi.

### FTIR characterization of SE oil degradation

**Figure 4:**
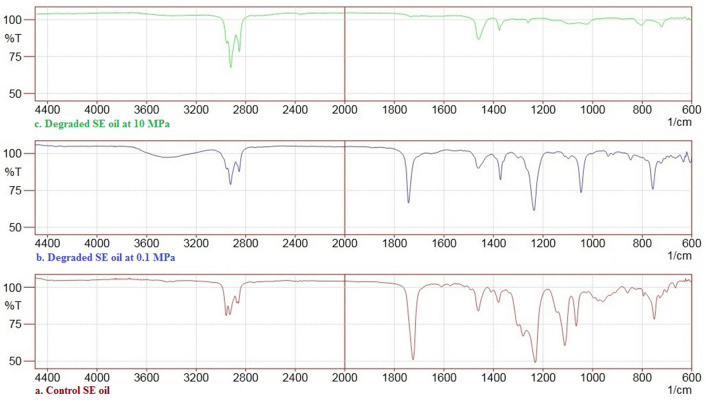
FTIR spectra analysis (**a**) SE oil, (**b**) degraded SE oil at 0.1 MPa and (**c**) degraded SE oil at 10 MPa pressure conditions.

The FTIR spectra of SE oil showed the presence of aliphatic stretches at 2900 and 2853 cm^−1^ (C–H) and aromatic stretching bands at 1500–1600 cm^−1^ (C=C). Peaks at the range of 1690–1720 cm^−1^, 1635–1655 cm^−1^ and 1500–1600 cm^−1^ were ascribed to the stretching vibration of C=O bond stretching vibrations, ketones and quinine molecules (Fig. 4a). The 0.1 MPa culture conditions induced major changes in its structural properties of SE oil and it is elucidated by the formation of major peak at 1000–1100 cm^−1^ (Fig. 4b). In 10 MPa conditions, many peaks disappeared and became less intense due to formation of degradation metabolites of SE oil. Additionally, few peaks in the range of 700–1500 cm^−1^ at both ambient and high pressure conditions correspond to the functional groups of phthalic acid, dicarboxylic acid and fatty acids were also detected (Fig. 4c).

### ^1^H NMR analysis of SE oil degradation

**Figure 5:**
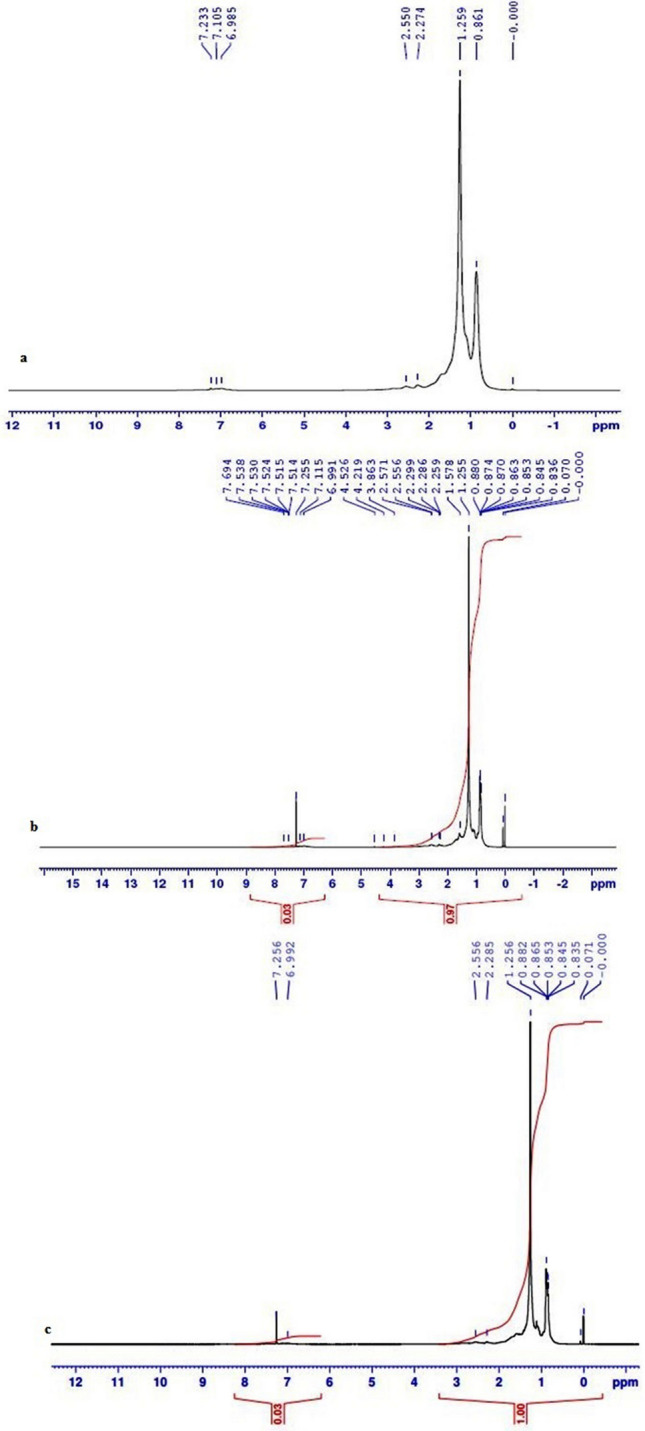
^1^H NMR analysis (**a**) SE oil, (**b**) degraded SE oil at 0.1 MPa and (**c**) degraded SE oil at 10 MPa pressure conditions.

The presence of peaks at 0–3 and 6.4–7.2 revealed the presence of substituted aromatics, heterocyclic aromatics and PAHs (Fig. 5a). The ^1^H NMR spectra of SE oil degradation metabolites at 0.1 and 10 MPa pressure by *A. sydowii* BOBA1 are shown in (Fig. 5b,c). In 0.1 MPa, the peak at 4.5, 4.2 and 3.8 ppm corresponding to the carboxylic acid proved the conversion of aromatic compounds by the fungal cells. In 10 MPa pressure, the total amount of aromatic molecule/group decreased, that indicated the decrease in overall integral of aromatic spectral region (Fig. 5c) as well as the variability in their chemical structure which was shown by significant increased number of signals in the spectra.

### ^13^C NMR analysis of SE oil degradation

**Figure 6:**
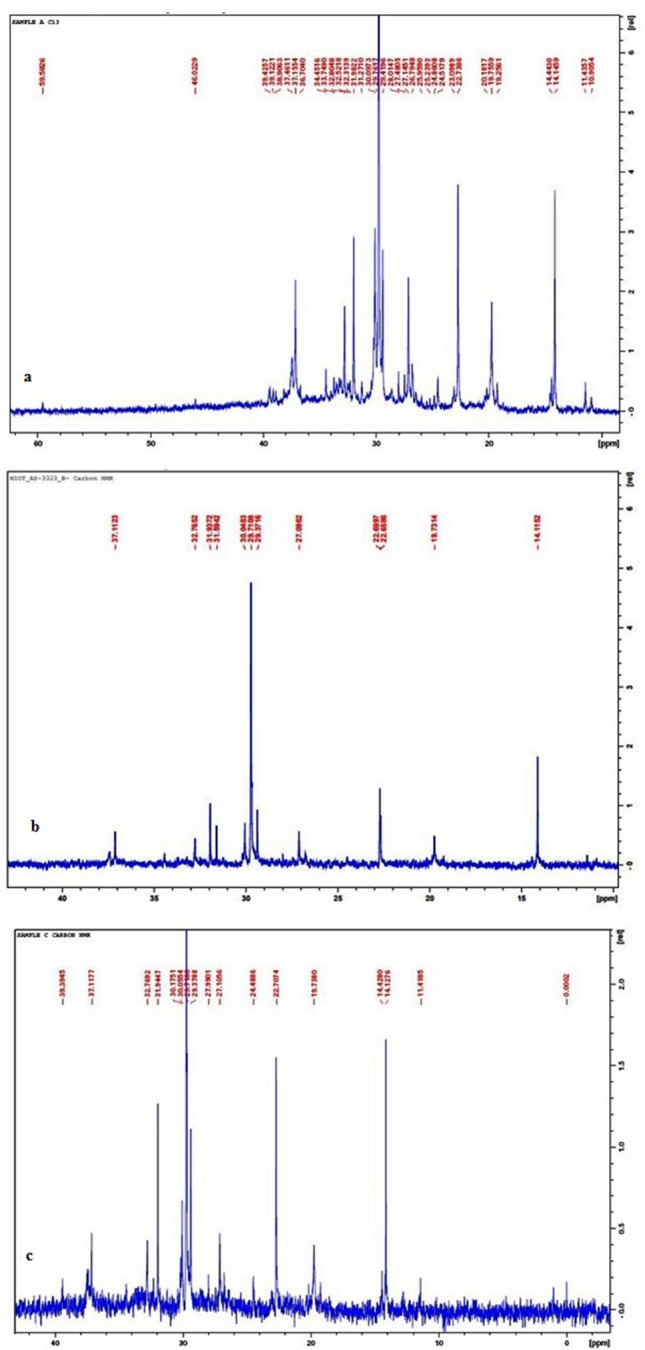
^13^C NMR analysis (**a**) SE oil, (**b**) degraded SE oil at 0.1 MPa and (**c**) degraded SE oil at 10 MPa pressure conditions.

The dominant signals in SE oil ^13^C NMR spectra corresponds to the presence of characteristic peaks at 10–30 ppm (CH_3_), 15–55 ppm (CH_2_), 25–60 ppm (CH) and 30–40 ppm (–C–) groups (Fig. 6a). In the spectra of degraded SE oil at 0.1 MPa, very weak signals of aromatic carbons (100–160 ppm) were observed (Fig. 6b). The sample from the 10 MPa conditions showed dominant modification in peaks ranging from 30 to 50 ppm (Fig. 6c).

### GC-MS characterization of SE oil degradation

The GC–MS analysis of SE oil showed the presence of PAHs, amines, ketones, carboxylic acids, siloxanes, steranes, diterpenes, triterpenes, fatty alcohols and epoxy groups (Table 3). Many refractory compounds were also identified in the SE oil which include phenanthrene, 8, 8′-Bi-2H-naptho[1,8-bc], 5H-cyclopropa (3, 4) benz (1,2-e, [2,2′-binaphthalene]-8, 8′-di, 9, 9′-biphenanthrene octacos, phenol, 2-methoxy-6-(3, 7, 11, difuro [2′, 3′: 5, 6: 3′, 2′: 7, 8, 1, 4: 5, 8-dimethanonaphthalene, spiro[9, 9′] difluorene-2, 2′-d and 2, 4-epoxymethanophenanthrene etc. The degradation of SE oil by *A. sydowii* BOBA1 at 0.1 and 10 MPa culture conditions resulted in breakdown of complex hydrocarbons after 21 days of incubation. The degradation of SE oil and the growth of BOBA1 did not modify the pH of the medium, which was found to be 6.07 ± 0.75 at the end of the experiment. The identification of carboxylic acid metabolites such as phthalic acid, decanoic acid, butenoic acid and acetic acid indicated the degradation of PAHs (Table 3). These metabolites were found to be absent in the GC-MS profile of fungal mycelium growth control (Supplementary Figure F1).

**Table 3:** GC–MS analysis of breakdown products of SE oil by *A. sydowii* BOBA1 at 0.1 and 10 MPa pressure conditions.

SE oil	RT	SE oil	RT
2,6-dimethyl heptadecane	6	2-Methoxy-6-(3,7,11,15,19,23,27,31-octamethyldotriaconta-2,6,10,14,18,22,26,30-octaenyl)phenol	47.9
Dodecamethylcyclohexasiloxane	6.1	17-Pentatriacontene	48
7-Heptadecene	7	9,12-Octadecadienoic acid	48.8
2,6-Dimethylnaphthalene	8.4	4,7-Benzofurandione,3-acetyl-3a,7a-dihydro-2-methyl-3a,5,6,7a-tetrakis[(trimethylsilyl)oxy]-	48.9
2,6,10-trimethyltetradecane	9	1-Hentetracontanol	49.3
Tetradecamethyl cycloheptasiloxane	9.4	N,N-Bis(2-hydroxyethyl)hexadecanamide	50.2
2,5-bis(1,1-dimethylethyl)-phenol	10	9,9′-Spirobi[fluorene]-2,2′-diol	50.5
Nonadecane	12	3,12-Oleandione	50.6
Hexadecamethylcyclooctasiloxane	14.4	2-Methoxy-6-methylphenol	54.3
2-Butyl-1-octanol	24	1,4:5,8-dimethano naphthalene	55
2-Hexadecanol	32		
Naphtho[2,3-b]thiophene	34.1	**Degradation metabolites**	**RT**
6-Methoxy-benzo[c]phenanthrene	36	**Products at 0.1 MPa**	
Benzenepropanoic acid	36.2	Dodecane	11.3
20-Oxo-3,9:14,15-diepoxypregnane	36.3	Benzothizole	11.6
4,5-Dimethylphenanthrene	36.7	Benzene	13.6
5-Methoxy-7-methylbenz(a)anthracene	38	Phthalic acid	29
Phenanthrene	38	Phthalic acid	37.6
Benzenamine	38.2	Decanoic acid	39.6
2-Bromo-1-octadecanol	38.4	Prosta-5,13-dien-1-oic acid	42.4
benzo(b)fluorene	39	2-Butenoic acid	45
Butanoic acid	41	Tetradecanoic acid	45.2
9-n-dodecylphenanthrene	43	Acetic acid	49.4
Stigmastane	45.7	**Products at 10 MPa**	
9-Desoxo-9-x-acetoxy-3-desoxy-7.8.12-tri-O-acetylingol-3-one	45.8	Dodecane	11.7
2-Amino-3-cyano-4-methyl-4,6-bis-(5-nitro-benzofuran-2-yl)-cyclohexa-1,5-dien-1,3-dicarboxylic acid, diethyl ester	45.9	Phthalic acid	29.6
2,2-Bis[4-[[4-chloro-6-(3-ethynylphenoxy)-1,3,5-triazin-2-yl]oxy]phenyl]propane	46.1	Decanoic acid	39.7
17-Pentatriacontene	46.1	2-Butenoic acid	45.4
4alpha-Phorbol 12,13-didecanonate	47.3	Tetradecanoic acid	45.3

### Genome properties and bioinformatics analysis

**Figure 7:**
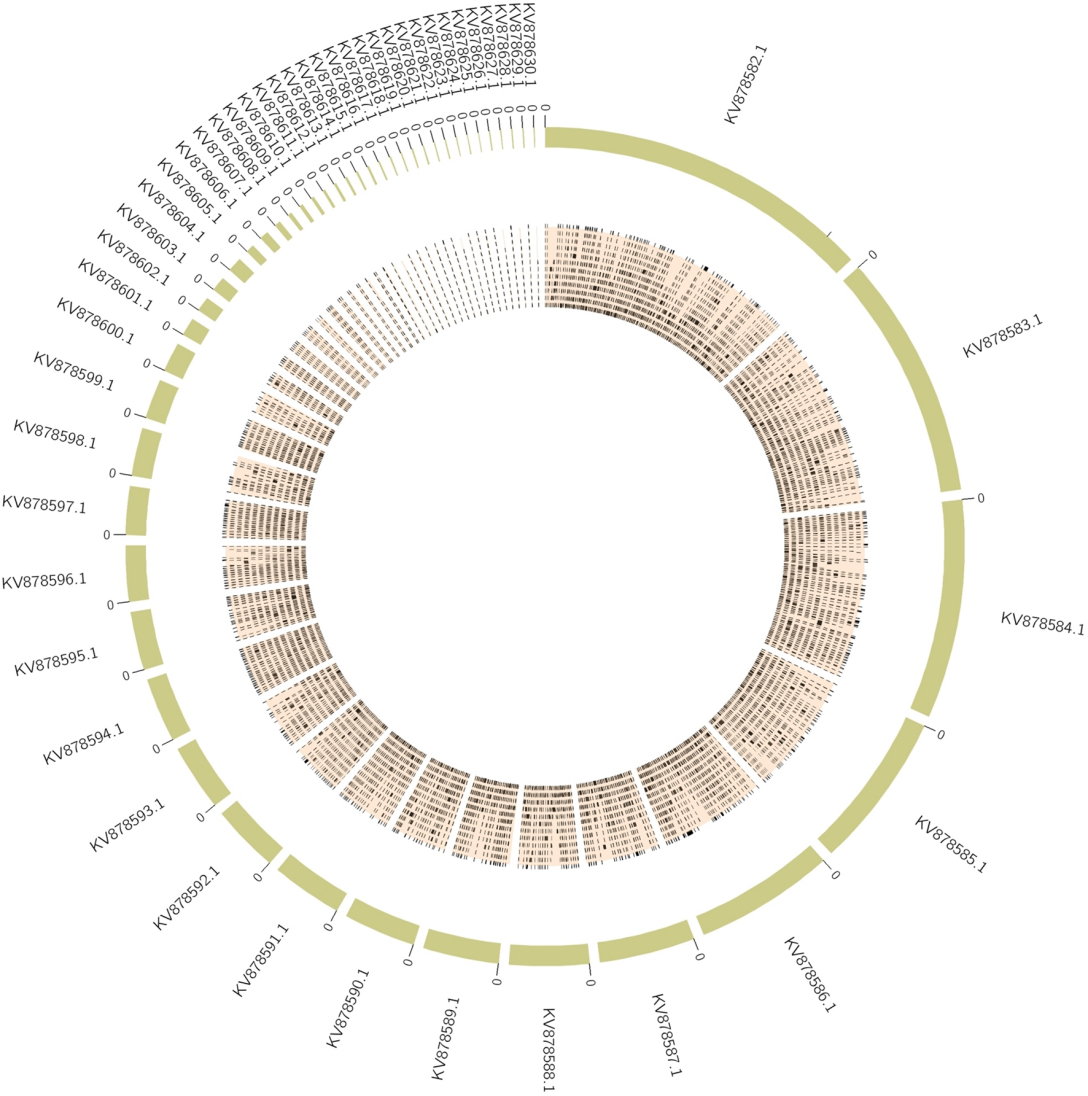
Circos plot of *A. sydowii* BOBA1 using ClicO (http://103.47.253.210:3000/home). The yellow green tiles represents the *A*. *sydowii* CBS 593.65 and the orange black bar shows the similarity of assembled genome contigs with the reference genome.

**Figure 8:**
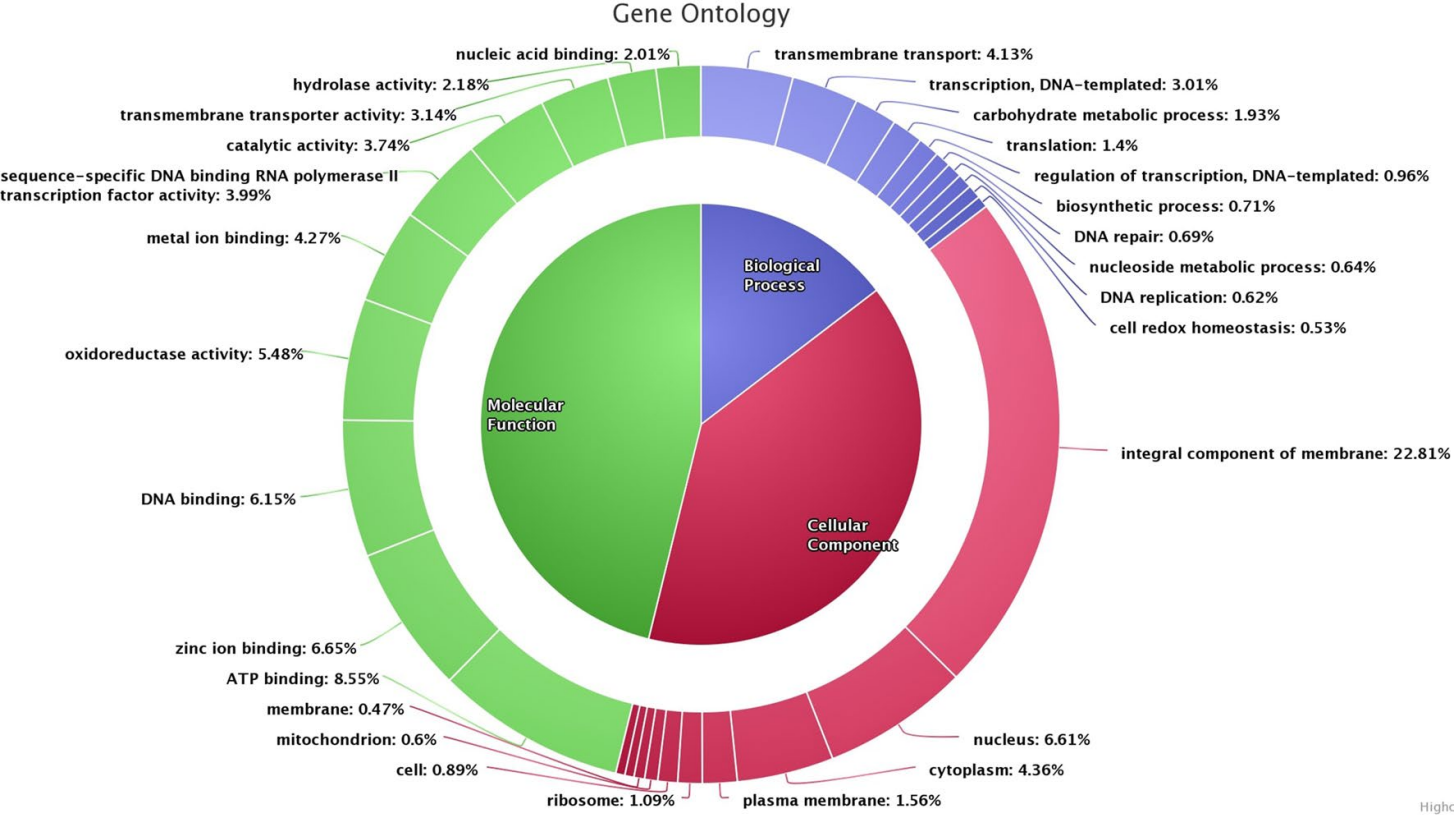
Cluster of orthologous group (COG) functional classification of genes in *A. sydowii* BOB A1 genome corroborate the presence of genes involved in biodegradation (oxidoreductase, catalytic and hydrolase protein) (https://www.highcharts.com/demo/pie-donut).

The assembled genome of the strain BOBA1 has a size of 38,795,664 bp widely distributed in 2582 scaffolds. A total of 18,932 genes were predicted; among these 16,247 and 2685 were identified as known and uncharacterized protein genes, respectively. Therefore the presence of coding protein genes was found to be 85.81% of the total genes (Table 4). Genome comparison of BOBA1 with CBS 593.65 revealed presence of more than 1400 unique genes (Supplementary Figure F2). The potential functions of unique genes were identified using UniProt (Supplementary data E1). The circular genome map of BOBA1 was aligned with *A. sydowii* CBS 593.65 by ClicO using circos and the coverage was nearly 89.94% of its length (Fig. 7). The yellow green tiles represent the *A*. *sydowii* CBS 593.65 and the orange black bars show the similarity of assembled genome contigs with the reference genome. The genome sequence was submitted in NCBI under the accession number WUTQ00000000. The gene ontology of BOBA1 validates the presence of genes involved in biological process (14.62%), cellular component (38.39%) and metabolic molecular function (46.16%) (Fig. 8). Presence of oxidoreductase (5.48%), catalytic (3.74%) and hydrolase (2.18%) activity in cluster of orthologous group analysis showed the ability of biodegradation and metabolic activities.

**Table 4:** Genomic features of the isolated *A. sydowii* BOBA1.

Features	Description
Size (bp)	38,795,664
Assembler	SPAdes v. 13.3.0
Number of scaffolds generated	2582
Maximal length, bp	354,018
Minimal length, bp	200
Average scaffolds length (bp)	2407
Median scaffolds length (bp)	279
**Total coding genes**	18,932
Function assigned	16,247
Uncharacterized protein	2685
N50 value	31,598
L50 value	330
Maximum percentage of Ns per sequence	0.01
Scaffolds ≥ 10 Kbp	1078
GenBank assembly accession	GCA_009828905.1

### Degradation of SE oil components

**Figure 9:**
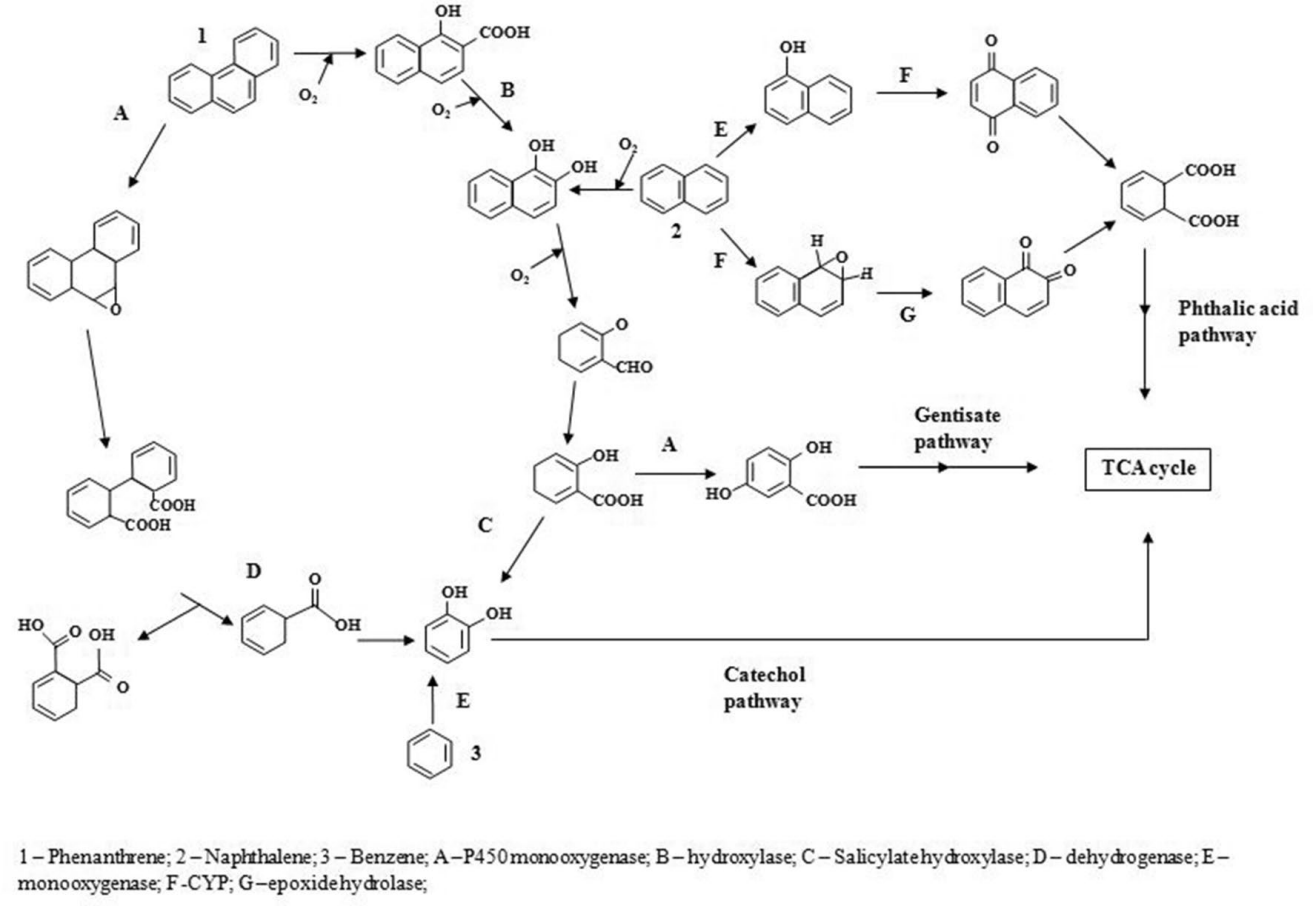
Proposed aerobic pathway involved during benzene, naphthalene and phenanthrene degradation in *A. sydowii* BOBA1.

Various types of petroleum hydrocarbons ranging from the most commonly known aromatic compound benzene to fused aromatic compounds like naphthalene and phenanthrene are generally degraded through one or more independent enzymatic systems. Possible degradation pathways of aromatic compounds were deduced following the combined analysis of whole genome annotation and GC–MS profile. The aerobic degradation and enzymes involved in transformation of benzene, naphthalene and phenanthrene are shown in Fig. 9. Phenanthrene was converted into phenanthrene 9, 10 oxide by cytochrome P450 monooxygenase through oxidation process. Metabolism of epoxide hydrolase and quinone reductase forms trans- 9, 10 phenanthrene dihydrodiol and diphenic acid, respectively^21^. Diphenic acid was reduced into 1, 2-benzene dicarboxylic acid and benzoate. These products were finally converted into CO_2_. In another plausible degradation pathway, phenanthrene was converted into 1-hydroxy-2-naphthoic acid and 1, 2-dihydroxynaphthalene by the action of hydroxylase. Subsequently forming salicylaldehyde by oxidoreductase, this in turn enters into the central pathway through catechol as an intermediate compound^22^. Naphthalene was converted into dihydroxy naphthalene by the action of multicomponent enzymes dioxygenase and dehydrogenase. Subsequently 1, 2-dihydroxy naphthalene was metabolized into salicylaldehyde through hydroxybenzalpyruvic acid. Salicylaldehyde dehydrogenase oxidizes salicylaldehyde into salicylate. This was decarboxylated to catechol by salicylate hydroxylase and can also gets converted into gentisate by salicylate 5-hydroxylase^23^. Finally, both catechol and gentisate intermediates undergo mineralization and enters into central carbon pathway. Epoxide hydrolase and cytochrome monoxygenase converts naphthalene into 1, 2 naphthoquinone and 1, 4-naphthoquinone, respectively. These quinone derivatives form phthalic acid by the action of ring opening hydrolases^24^. Benzene was converted into phenol and catechol by successive hydroxylation leading to the formation of TCA intermediates.

### Genes involved in hydrocarbon biodegradation pathways

The ability of *A. sydowii* BOBA1 to degrade PAHs and xenobiotics is supported by the identification of genes involved in the biodegradation of n-alkanes and aromatics, including cytochrome P450 alkane hydroxylase, cytochrome P450 benzoate 4-monoxygenase, salicylate hydroxylase, dioxygenases, succinate dehydrogenase, and catechol 1, 2-dioxygenase. The present study help in elucidating the mechanisms involved in myco-degradations (Supplementary Tables S1–S9).

### Cytochromes

Marine fungi have developed multiplex enzymatic machinery for degradation of complex toxic compounds from the environment. The presence of cytochrome P450 monoxygenases (CYP1) and glutathione transferases genes revealed the catabolic abilities of *A. sydowii* BOBA1 to degrade environmental contaminants.

### Peroxidases

A group of peroxidase enzymes, such as thiol peroxidase (EC 1.11.1.15), catalase-peroxidase (EC 1.11.1.21), peroxiredoxin (EC 1.11.1.15), deferrochelatase peroxidase (EC 1.11.1.-), heme-haloperoxidase (EC 1.11.2.1), glutathione peroxidase (EC 1.11.1.9) and peroxidase (EC 1.11.1.7) with high redox potential confirmed the biodegradative capability of *A. sydowii* BOBA1 towards a variety of complex aromatic hydrocarbons and recalcitrant xenobiotics.

### Glutathione system

This system acts as a major cellular redox buffer catalyzing major enzymatic activities. Several studies have shown the functional roles of various classes of enzymes involving glutathione. Homologs of the enzyme such as glutathione peroxidase, glutathione s-transferase, hydroxyacylglutathione hydrolase, glutathione reductase, lactoylglutathione lyase, gamma-glutamylcyclotransferase, glutathione synthetase, glutathione dehydrogenase and S-formylglutathione hydrolase encoding genes involved in aromatic biodegradation were observed.

### Xenobiotic detoxification genes

The genomic analysis shows the ability of filamentous fungi *A. sydowii* BOBA1 to metabolize xenobiotics. The xenobiotic compounds were detoxified and tranformed through coordinated mechanisms of cytochrome P450 monoxygenases, epoxide hydrolases, aryl sulfotransferase, glutathione S-transferase, UDP-glucosyltransferase, N-acetyltransferase and ATP-binding cassette [ABC] efflux transporters.

### Piezophilic adaptation

*A. sydowii* BOBA1 genome analysis revealed that adaptation in deep-sea involves a diverse array of genes such as d-alanine-d-alanine ligase, alanine dehydrogenase, S-adenosyl methionine (SAM) methyltransferase, cytochrome *c*, Uvr genes and alkylhydroperoxide reductase (Supplemetary Table S10).

## Discussion

To the best of our knowledge, this is the first study on deep-sea piezotolerant fungus *A. sydowii* BOBA1 degrading SE oil at in situ deep-sea conditions and analysis of the whole genome sequences for potential mycoremediation applications in extreme marine environment. Microbial mediated remediation technologies are eco-friendly and cost-effective for the remediation of toxic compounds. Despite the significant role of predominant genera, variable combinations of bacterial and fungal networking with complementary catabolic competences could be involved in complete biodegradation of these pollutants. In the last decade, fungal diversity in deep-sea environments have been investigated extensively by culture-dependent and culture-independent metagenomic approaches^25,26^. The presence of fungi in oil-contaminated marine sediments are often reported and predominant shift in eukaryotic microbial structure in post-oil spill sediment has been observed in the Indian marine environments^27^. However, the knowledge on fungal diversity associated with hydrocarbon and xenobiotic compound degradation in deep-sea environments have not been explored extensively. The aim of this study was to investigate the metabolic role of fungal biodegradation of SE oil in deep-sea conditions.

Some fungal species play a significant role in the PAH degradation processes, results from this research indicated that this deep-sea isolate was an effective strain to degrade crude oil, engine oil, n-hexadecane, kerosene, petrol, phenol, toluene and xylene (Table 1). The fungal isolates showing high degradation capability was selected as best strain for extensive studies. The growth and activity of BOBA1 was stable in the presence of surfactants such as sodium dodecyl sulphate and tween 80. Often bioremediation applications depend on the use of surfactants which increase the solubility and bioavailability of PAHs^28^. Specific interactions between the microorganisms and surfactants play an important role in biodegradation and bioremediation. It was proposed that in presence of surfactant, the surfactant-compatible BOBA1 may influence the biodegradation of different PAHs to different degrees. High diversity of deep-sea fungi have acclimatized and evolved multitude of mechanisms to grow and play an active role in extreme environment of different pH, salinity, pressure and temperature^29^. The *A. sydowii* BOBA1 could grow in the pH range of 4.0–10.0, NaCl concentration of 1–10% (w/v) and temperature range of 10–40 °C, demonstrating its capability to grow and catalyze diverse function at broad environmental conditions (Table 2). It was found that fungus posses adapted mechanisms to tolerate wide test concentrations of SE oil and utilize hydrocarbons as sole carbon source. Numerous studies have reported that different environmental factors influence hydrocarbon degradation mechanisms in marine fungi. Thus the ability of the isolate to grow at variable conditions is vital in removal of PAHs and xenobiotics from extreme marine environments.

The RB was selected as carrier matrix for the strain BOBA1 immobilization because it is readily available, cost effective and eco-friendly. The SEM analysis revealed intensive and deeply penetrated mycelia structure of fungal cells on RB (Fig. 1). The critical role of agro residues was to enable cells to adhere and maintain the integrity of the fungal cells. Agro residues based supporting material has large porosity which permits remarkable mass transfer of carbon and nutrient sources coupled with oxygen^30^. Mild alkali pre-treatment of RB and washing could remove the amorphous material which makes the fibre surface rough with increased crystallinity index^31^. The crystallinity degree of cellulose affects the easy access of hydrolytic enzymes to polymeric molecules^32^ making RB as suitable immobilization carrier. The adhesion properties of *A. sydowii* BOBA1 enable to aggregate and colonize effectively on RB. Thus the filamentous fungus immobilized on a pre-treated natural carrier material was confined to remove or mitigate the potential pollutants. Our earlier studies have proved immobilization carrier for bioremediation application should be non-toxic and readily degradable^33^. Thus pre-treated RB was opted for immobilization and biodegradation application. Furthermore, it has been reported that the immobilized fungus showed a higher removal of PAH than the free cells^34^. Immobilization of *Aspergillus* to natural carrier materials drastically increased the cell concentration. Immobilized biomass reached about 28.76 mg drycells/g RB. This would make the bioprocess convenient and efficient. These findings substantiated the use of RB as a suitable immobilization matrix for fungal immobilization. The concept of mycoremediation by immobilizing deep-sea fungus in lignocellulose materials, which is readily degradable and non-accumulant in the environment is more advantageous than the other conventional methods. Studies on N and P fertilization confirmed that supplementation of the CO(NH_2_)_2_ and (NH_4_)H_2_PO_4_ resulted in enhanced levels of oil degradation. The result of this study has already established very well the relationship between the marine fungi, N and P source in oil degradation. These finding concurs with our earlier reports on the effect of CO(NH_2_)_2_ and K_2_HPO_4_ in biodegradation of crude oil by immobilized bacterial consortium^33^.

High pressure has a positive influence in deep-sea microorganisms. The SE oil degradation by *A. sydowii* BOBA1 was studied at 0.1 MPa and 10 MPa pressure conditions. Interestingly, initial lag phase around 0–7 days were required for the fungal cells to adapt to higher concentration of SE oil containing high concentrations of PAHs and to exhibit oil degradation efficiency. The average net removal rate of SE oil was high on 21 days and reached maximum at 28 days. This confirmed two phase degradation by deep-sea *A. sydowii* BOBA1, maximum degradation was observed in phase I (14–21 day) where both the degradation and biomass were high (Fig. 2). The growth of filamentous fungi stabilized thereafter and follows stationary phase (Fig. 2). This was the phase II, where consistent degradation was observed (21–28 day). Based on these findings, 21 days of incubation time was selected as a time required for degradation of complex aromatic hydrocarbons present in SE oils. In this period, much higher levels of microbial activity were observed, probably due to physiological adaptation to the cultivation conditions. This proves that the fungi have the potential to release highly active hydrocarbon-degrading myco-enzymes within a short period^35^. In one of the research study, the potential of white rot basidiomycetes in degradation of bunker fuel oil with saw dust as an immobilization matrix was demonstrated^34^. These results suggested that the, addition of agro residues can favour and promote the microbial growth and is a possible way to enhance bioremediation strategy. Moreover the use of deep-sea fungi to degrade PAHs is underexploited; our results on *A. sydowii* BOBA1 degrading SE oil are much relevant for marine oil spill bioremediation applications. Previous reports were available about the possible role of *Aspergillus* sp*.* in mycoremediation of xenobiotic compounds^20^. Besides the PAH degrading fungi reported in the present study, ascomycete fungi *Aspergillus* sp. are known to degrade polyurethane compound^36^.

The complication in carrying out high pressure studies is well known, and analysis on PAHs degradation by deep-sea fungi is limited. In this study we demonstrated the degradation of SE oil by deep-sea *A*. *sydowii* BOBA1 and showed that it is negatively affected by pressure. The morphology of the fungi changed at 10 MPa pressure conditions, the cells were more aggregated and reduced in size with an average hypha filament diameter of 0.10–0.13 µm (Fig. 3). Mycelial aggregation occurs due to electrostatic and hydrophobic interactions. These findings elucidated that *A*. *sydowii* BOBA1 may play a greater role in hydrocarbon degradation in both shallow and deep-sea environments. Earlier studies of our lab have reported a synchronous negative effect of high pressure conditions on the growth of deep-sea *Nesiotobacter exalbescens* COD22^37^ and *Bacillus subtilis*^38^. Moreover, we have reported ability of fungus to respond to the changing environmental signals by altering their morphological or physiological state. The size of branching hyphae of deep-sea fungus *Nigrospora* sp*.* got reduced considerably when it was cultured at 100 bar pressure^39^. This significant morphological change confirmed the pressure tolerance and elucidated how varying pressurized environment induced changes in the growth of fungus. Metabolic activity of *A*. *sydowii* BOBA1 was found to be high at 10 MPa culture conditions. The influence of pressure decreased the size of cells which may significantly increase the surface area of active sites resulting in high rates of degradation. Our earlier finding correlated with this result showing the impact of pressure on enhancing the degradation of toluene by deep-sea bacteria at 100 bar pressure^37^. Apart from the cell morphology, piezophilic adaptation to elevated hydrostatic pressure conditions involves major cellular and molecular processes^40^.

*A. sydowii* BOBA1 displayed higher percentage of SE oil degradation. The decrease in the BOD of treated samples indicated the reduction in the organic load and confirmed the SE oil degradation efficiency at both ambient and elevated pressure conditions. The breakdown products of complex hydrocarbons and recalcitrant organic compounds present in the SE oil by deep-sea fungus *A. sydowii* BOBA1 at 0.1 and 10 MPa were characterized by FTIR, NMR and GC–MS spectroscopic analysis. In FTIR, the peaks observed at 2800–3000 cm^−1^, 1500–1600 cm^−1^ and 1350–1450 cm^−1^ in control SE oil have been assigned to hydrocarbon mixture having C=O and C–H stretching vibrations. Minor peaks in the range of 2350–2360 cm^−1^ corresponds to C–H stretching of alkenes. A band at 720 cm^−1^ is associated with long straight-chain groups of alkanes. The strong peaks at 1370–1455 cm^−1^ can be attributed to C–H deformation in methyl groups (Fig. 4a). The FTIR spectrum of treated sample at 0.1 MPa indicated the formation of peaks corresponding to the functional groups of dicarboxylic and fatty acids (Fig. 4b). The peaks at 1380, 1450 and 1700 cm^−1^ revealed the presence of C=O, C–O and O–H stretching vibrations of carboxylic acids^41^. In 10 MPa conditions, many peaks disappeared and became less intense, indicating that PAHs and aromatics were completely biodegraded (Fig. 4c). Stretching vibrations peaks of Fig. 4c showed the molecular disorientation of aromatic and aliphatic molecules, and confirmed the formation of carboxylic acids through the oxidation of hydrocarbons and complex aromatics by distinctive extracellular enzymes of microbial origin^42^.

The ^1^H NMR spectrum of SE oil showed dominant peaks at 0–3 chemical shifts (δ). These intense peaks indicated the presence of aliphatic protons. The dominant signals in the spectra correspond to the protons in the CH_2_ groups of alkyl-type chains and CH_3_ end groups (Fig. 5a). Compounds with ^1^H chemical shifts at 4.5, 4.2 and 3.8 ppm were observed to accumulate during fungal growth at 0.1 MPa (Fig. 5b). These protons may be assigned to the presence of carboxylic group^43^. The major difference was the disappearance of peaks at 7.2, 2.3 and 1.5 and it corresponds to the breakdown of alkyl and aromatic ring compounds (Fig. 5c). The ^13^C NMR spectra signals and their chemical shift values at 10–30 ppm (CH_3_), 15–55 ppm (CH_2_), 25–60 ppm (CH) and 30–40 ppm (–C–) groups were influenced by biodegradation. The SE oil contains a variety of different saturated and unsaturated alkyl chains varying in size and their chemical structure (Fig. 6a). Although the SE oil underwent a variety of changes during the biodegradation process, it has a large number of CH_2_ groups and CH_3_ end groups. In the spectra of degraded SE oil at 0.1 MPa, very weak signals in aromatic carbons (100–160 ppm) were observed. This confirmed the presence of low aromatic content even after degradation. Mild chemical shift between 70 and 80 ppm could be due to microbial-oxidation of hydrocarbon (Fig. 6b). The sample from the high pressure grown cells showed peaks from 30 to 50 ppm ranges (Fig. 6c). The results of ^13^C NMR analysis demonstrated the breakdown of aromatic carbons by cells grown at 10 MPa by microbial degradation^44^.

The mixture of n-hexane and dichloromethane were demonstrated to be effective in removing hydrocarbons from contaminated samples^45^. In our earlier studies, the ratio effect between the solvent and samples on the extraction efficiency was discussed^33^. Table 3 showed the presence of PAHs, alkanes, alkenes, amines, ketones, carboxylic acids, siloxanes, steranes, diterpenes, triterpenes, fatty alcohols and epoxy groups in the SE oil. The percentage of major constituents include 8.5% of alkanes, 7.5% of aliphatic hydrocarbon, 1.5% of aromatic hydrocarbon, 1.2% of alkenes and 0.25% of siloxanes and epoxy groups. Further these results may be attributed to the combined effect of factors such as viscosity, chemical additives, concentration, density and solubility parameters. *A. sydowii* BOBA1 was able to assimilate the major constituents at 0.1 MPa pressure. Metabolism of unsaturated, branched chain aliphatics and PAHs via hydroxylation and oxidation by fungal cells led to the accumulation of epoxides, alcohols, diols and carboxylic acids^46^. At 10 MPa pressure, the presence of phthalic acid, decanoic acid, butenoic acid and acetic acid confirmed the complete breakdown and assimilation of complex hydrocarbons. The degradation rate reached 60 to 75% in 0.1 MPa and 70 to 85% at 10 MPa pressure conditions, respectively. The GC–MS analysis revealed, at both ambient and elevated pressure conditions the fungal cells exerted high degree of degradation towards alkane, alkene and PAHs. Table 3 indicated the breakdown metabolites and converted products formed due to mycodegradation. After 21 days of *A. sydowii* BOBA1 treatment, the dominant 2, 3 and 4 ringed PAHs were completely mineralized. Subsequent enzymatic catalytic reactions and ring cleavage mechanism of each aromatic compound led to the accumulation of ring-fission phthalic acid as principal metabolite. The accumulation of phthalic acid and phenol elucidated the oxidative degradation of PAHs. The most abundant PAHs breakdown products were identified as phthalic acid and this has been reported in phenanthrene degradation^47^. Since the accumulation of phthalic acid was not evidenced it can be concluded that complex aromatics were completely mineralized by immobilized cells. The result matched with the potential pathway proposed by different researchers for aerobic degradation of phenanthrene, fluoranthene and naphthalene. The PAH oxidation by white-rot fungus, *Anthracophyllum discolor* produced more metabolites such as anthraquinone, phthalic acid and 4-hydroxy-9-fluorenone^48^. White rot fungus, *Pleurotus pulmonarius* was found to follow phthalic acid pathway in the process of fluoranthene degradation^49^. Overall, the results reported here demonstrated the biodegradation potential of *A. sydowii* BOBA1 for the use of mycoremediation of PAHs contamination.

The degradation of hydrocarbon contaminants was found to be strongly dependent on the genus of the fungi. *Aspergillus* sp. are the most dominant fungi of the marine ecosystem having various ecological roles^50^, frequently prevails in contaminated sites and can metabolize certain PAHs^51^. A strong correlation between the denitrification and hydrocarbon degradation confirmed that denitrifying microbes could play a major role mineralization of PAHs under anoxic conditions^52^. Microbes derive energy through energy-yielding biochemical reactions mediated by these enzymes to cleave chemical bonds and assist transfer of electrons from a reduced organic substrate (donor) to another chemical compound (acceptor). Hence it is very important to investigate the role of this group of enzymes in crude oil biodegradation. The comparative genome map of BOBA1 with CBS 593.65 strain from KV878582.1 to KV878601.1 revealed several shared coding sequences involved in DNA replication, transcription, cell cycle control, cell division and nucleotide transport metabolism (Fig. 7). The analysis of gene ontology revealed the presence of 46.16% genes involved in metabolic molecular function (Fig. 8). The genomic analysis of filamentous fungi *A. sydowii* BOBA1 showed the presence of enzymes that metabolize PAHs, aliphatic hydrocarbon and xenobiotic compounds. Several unique genes (1482) putatively associated with cellular and metabolic pathways were detected in *A. sydowii* BOBA1 (Supplementary Figure F2 and Supplementary data E1). The functional annotation of these unique genes revealed the presence of high catalytic enzymes such as monooxygenase (4), dioxygenase (6), glutathione transport system (4), peroxidase (3) and semialdehyde dehydrogenase (1) that were actively involved in the degradation of aromatic hydrocarbons.

Biodegradation/mineralization of aliphatic and aromatic hydrocarbons may occur under anaerobic or aerobic conditions. Through oxidation (availability of O_2_ as an electron acceptor) by monooxygenases (Supplementary Table 1) and dioxygenases (Supplementary Table 2) the catabolism of hydrocarbons was rapid^53^. Dioxygenases primarily oxidize aromatic compounds, and therefore, have application in environmental remediation. *A. sydowii* BOBA1 mineralizes the aromatic hydrocarbons benzene, phenanthrene and naphthalene that are present in SE oil under aerobic conditions. Metabolic enzymes that catalyze xenobiotic biotransformation and detoxification reactions are classified as phase I and phase II enzymes. Cytochrome P450 monoxygenase and epoxide hydrolase (Supplementary Table 3) constitute two important phase I oxidation enzyme groups. Phase II reactions are catalyzed by glutathione S-transferase (Supplementary Table 4), aryl sulfotransferase (Supplementary Table 5), NAD(P)H quinone oxidoreductase and UDP-glucuronosyltransferase. Fungal enzymes play an important role in the degradation of aromatic hydrocarbons than bacteria^19^. *A. sydowii* BOBA1 detoxifies and tranforms aromatic hydrocarbons through coordinated mechanisms of cytochrome P450 monoxygenases, epoxide hydrolases, aryl sulfotransferase, glutathione S-transferase, UDP-glucosyltransferase, N-acetyltransferase and ATP-binding cassette [ABC] efflux transporters. Bioremediation of PAHs by non-lignolytic fungi is done with two phases. Phase I is initiated by P450 and epoxide hydrolases with the oxidation of the aromatic hydrocarbon. Conversion of toxic compounds usually requires molecular structures to pass through cell walls which are further converted by cell membrane bound enzymes such as P450s or epoxide hydrolases. The fungal cytochrome P450 system can serve as versatile catalyst for region and stereospecific oxidation of hydrocarbons, and can be ideal substitutes for chemical catalysts^54^. Fungal cytochrome P450 systems were reported to be involved in metabolisms of signaling, biosynthesis, detoxification and degradation^55^. Separate iso-forms of cytochrome P450 were found for their capabilities for specific oxidations^56^. Specific fungal cytochrome P450 monoxygenase from *Phanerochaete chrysosporium* oxidized recalcitrant fused ring high molecular weight PAHs^57^. Further in phase II through intracellular catabolism by glutathione S-transferases, oxidoreductases and transferases the aromatic compounds were further reduced into TCA intermediates^58^. Glutathione system plays an important role in degradation of xenobiotic compounds^4^. Fungal specific class A glutathione transferase was reported in saprotrophic fungi^59^. High level of the S-transferase activity in *Stenotrophomonas maltophilia* strains exhibited higher biodegradation efficiency and might have initiated the reaction of biological degradation through glutathione S-transferase enzyme by activation of the metabolic pathways involved in biotransformation of the creosote hydrocarbons^60^.

It is important to note that in aerobic pathway hydroxylation or dioxygenation reactions convert a large spectrum of hydrocarbons resulting in the formation of vicinal dihydroxylated benzene derivatives. The common intermediate compounds produced includes catechol (1,2-dihydroxybenzene), hydroxyquinol (1,2,4-trihydroxybenzene) and protocatechuate (3,4-dihydroxybenzoic acid). These intermediates undergo ring cleavage and resulting metabolites enter into central metabolism^53^. The strain BOBA1 follows ortho cleavage for the degradation of PAHs. Catechol is an important intermediate in the aromatic compound degradation pathway. Catechol is further oxidized into cis,cis-muconate and follows β-ketoadipate pathway. Strain BOBA1 carries various genes for aromatic hydrocarbon degradation via β-ketoadipate pathway, which included 3-carboxy-cis,cis-muconate cycloisomerase, 4-carboxymuconolactone decarboxylase, β-ketoadipate succinyl CoA transferase and β-ketoadipyl CoA thiolase. The initial ring cleavage of PAHs is similar for catechol and gentisate pathway. Salicylic acid is converted into either catechol or gentisic acid by the action of salicylate hydroxylase. Salicylate 1-hydroxylase converts salicylic acid into catechol whereas salicylate 5-hydroxylase converts it into gentisic acid. Further 1,2 dioxygenase is involved to form maleyl pyruvate, isomerase and hydrolase mineralizes to convert it into fumarate and pyruvate that enters into central metabolic pathway.

*A. sydowii* BOBA1 possess diverse peroxidase systems with distinct functional specificity to catalyze biodegradation process. Peroxidases (Supplemetary Table 6) are an important class of oxidoreductases that catalyse a variety of reactions such as reduction and oxidation of a broad range of complex compounds. The low functional specificity and high redox potential of peroxidases enable the oxidation of a broad range of aromatic hydrocarbons and other recalcitrant contaminants. PAHs are oxidized by an array of peroxidases like lignin peroxidase and manganese peroxidase. The fungus *Lasiodiplodia theobromae* isolated from a PAHs polluted soil showed enhanced lignin peroxidase activity during benzo[a]pyrene degradation^61^. Protocatechuate 3,4-dioxygenase alpha subunit and beta subunit, which are found in the genome of BOBA1, were reported to be involved in benzoate and xylene degradation. Aerobic ring cleavage of protocatechuate is catalyzed by protocatechuate 3,4-dioxygenase to generate 3-carboxyl-cis,cis-muconate which is further converted into 4-carboxymuconolactone by 3-carboxy-cis,cis-muconate cycloisomerase. Further it follows β-ketoadipate pathway for the degradation. A UDP-glucosyltransferase family and arylsulphotransferase enzymes are xenobiotic metabolizing enzymes, which might be essential in the detoxification of PAHs and their degradation products. Vanillin dehydrogenase belongs to the aldehyde dehydrogenase family (Supplementary Table 7). It is involved in converting xylene to methyl benzoate and toluene to benzoate respectively. Two copies of gene encoding for 2-hydroxy-6-oxo-6-phenylhexa-2,4-dienoate hydrolase was present in strain BOBA1 genome, which may be responsible for the degradation of biphenyl or polychlorinated biphenyl. The genomic analyses of deep-sea *A. sydowii* BOBA1 confirmed the presence of abundant and diverse enzymatic systems to metabolically utilize complex PAHs and xenobiotic compounds. These findings were fully consistent with the data on sequencing and profiles of enzymes (Fig. 9).

Microbial siderophores have been known to facilitate the PAHs degradation activities through a vital mechanism, by facilitating the supply of Fe to participating microorganisms under Fe-limiting conditions^62^. Preliminary genome annotation of *A. sydowii* BOBA1 allowed the identification of potential siderophores (Supplementary Table 8). The presence of NADPH siderophore reductase, siderophore ABC transporter, siderophore receptor, ferrichrome receptor FCoA, ferrochelatase and deferrochelatase confirmed the functional role of hydroxamate ferrichrome type siderophores in *A. sydowii* BOBA1. The intradiol cleaving key enzymes utilize Fe(III), whereas the extradiol cleaving enzymes utilize Fe(II) and Mn(II). The siderophore producing microorganisms have an advantage over the non-siderophore producing microorganisms by iron facilitation during hydrocarbon and metal bioremediation in marine ecosystems^63^. *Aspergillus* sp. use chemically diverse siderophores with diverse function to uptake, transport and storage of iron, further these chemical chelators coordinate and activate various key pathways^64^. It is interesting to note that *A. sydowii* BOBA1 possess siderophores mediated mechanisms which can perform wide array of functions to degrade PAHs and environmental contaminants associated with metals and metalloids. Rieske non-heme iron dependent oxygenases are useful to catalyze a wide variety of reactions in the degrading xenobiotics^65^. The crystal structure of naphthalene dioxygenase has confirmed the presence of a Rieske (2Fe-2S) cluster and mononuclear iron in each alpha subunit. It is interesting to note that the genes coding for rieske protein could initiate the oxidative degradation of aromatic compounds through specific dihydroxylation catabolic process. These probable applications of Rieske oxygenases in BOBA1 (Supplementary Table 9) may catalyze a wide variety of oxidative transformations in removal of persistent and toxic aromatic compounds. Moreover, it is clear from recent discoveries that the catalytic role of Rieske clusters extends far beyond the PAH degradation pathway and are known to play biological role in a variety of oxidative transformations^66^. Furthermore, BOBA1 showed genes that could facilitate tolerance to high pressure (Supplementary Table S10). Numerous genes or gene clusters to cope with extreme living conditions such as genes for d-alanine-d-alanine ligase (2) involved in peptidoglycan formation, alanine dehydrogenase (3) for NADH/NAD^+^ homeostasis and SAM domain containing protein (1) for tRNA modification were present in BOBA1^67^. The existence of cytochrome *c* (3) genes could provide more ability to cope with the high pressure environments^68^. BOBA1 contains high number of Uvr genes (10) involved in DNA repair mechanism^69^ and alkylhydroperoxide reductase (3) protecting the organism from multiple abiotic stresses^70^. These specific features should contribute for the metabolism of aromatic compounds and adaptation of BOBA1 in deep-sea environments.

## Materials and methods

### Sediment collection and isolation of oil-degrading fungi

Surface sediment samples were collected at a depth of 3000 m (13º21.527′N/80º53.077′E) from the Bay of Bengal, India, using Oceanographic research vessel Sagar Nidhi. The possibility of external contamination is high therefore careful precautionary measures were taken during the sampling process. The sediment samples were collected using sterile plastic syringe (50 ml) from the mid-point of the core immediately after the retrieval of the sediment cores. The enrichment was carried out with 50 g of sediment sample supplemented with 0.1% sterile engine oil prepared in 950 mL of enrichment medium containing NaCl 1%, KCl 0.1%, K_2_HPO_4_ 1%, (NH_4_)_2_SO_4_ 0.75% and peptone 0.1%. All the sub-samplings for isolation of hydrocarbonoclastic fungus were carried out inside the laminar flow hood in a laboratory on the research vessel. The fungal strains were isolated from enrichment medium on MSM agar plates supplemented with 0.1% (v/v) sterile engine oil. The SE oil was obtained from a private mechanical engineering work station at Chennai, Tamil Nadu, India.

### Selection of potent fungi from the isolates

From the isolates, the potent strain has to be selected based on the substrate specificity assay. Different hydrocarbon substrates (0.1%) such as brij-35, cedar wood oil, clove oil, crude oil, diesel, n-hexadecane, kerosene, petrol, phenol, silicone oil, sodium dodecyl sulphate, SE oil, toluene, triton X-100, tween 80 and xylene were added onto mineral salt medium and inoculated with the test isolates. The growth was monitored by measuring increase in cell density. The plates were incubated at 28 °C for 120 h and cell growth was monitored every 24 h.

### Molecular characterization

Molecular identification was carried out by using ITS1-ITS4 primers (forward 5′ TCCGTAGGTGAACCTGCGG 3′ and reverse 3′ TCCTCCGCTTATTGATATGC 5′). The amplified PCR products were purified and sequenced using automated sequencer. The obtained DNA sequences were compared to the reference sequences within the NCBI gene database using NCBI blast. The selected sequences were checked and further aligned using MEGA tool. The phylogenetic tree was generated using neighbor-joining method along with evolutionary distance.

### Growth physiology of fungus

The growth of fungus strain was investigated in mineral salt medium (MSM) g/L composed of MgSO_4_ (0.2), CaCl_2_ (0.02), KH_2_PO_4_ (1), K_2_HPO_4_ (1), FeCl_2_ (0.05) and NH_4_NO_3_ (1.0) with 0.1% SE oil at different pH (4.0–10.0), temperature (10–40 °C) and NaCl concentration (1–10% w/v). The viability of fungus was enumerated by plate count method on potato dextrose agar (BD, Difco). Effect of SE oil concentration was studied in MSM with 0.1, 1, 2, 5 and 10% SE oil. The tolerance of fungus to the different pH, temperature, NaCl and SE oil concentrations were analyzed.

### Immobilization and fungal growth analysis

RB (100 g) was boiled in 1L of 1% NaOH and washed with distilled water to bring down to neutral pH. The pretreated immobilization materials were dried and autoclaved separately before the start-up of experiment. The immobilization of cells was carried out in a flask containing 10.0 g of sterilized RB, 1L of MSM culture medium and 10% of strain BOBA1 cell suspension. These inoculums were incubated at 25 ± 2 ºC for 120 h at 200 rpm. The immobilized BOBA1 cells were collected and washed with sterile saline solutions to remove residual free cells. The dry weight of the cells immobilized on the matrix was measured by comparing the dry weight of the agro residues before and after immobilization. To determine the total viable count (TVC) of fungal cells at immobilized conditions, the RB was mixed in phosphate buffer and vortexed. The released cells were collected by centrifugation followed by cell mass measurement by standard plate count method using potato dextrose agar (BD, Difco). The cell counts were performed in duplicates and expressed in CFU/g of immobilized RB and CFU/mL of free cells. The surface morphology of the fungal cells immobilized on RB was analyzed using scanning electron microscope (JSM-IT500, JEOL, Japan).

### Degradation studies

The immobilized BOBA1 cells (2%) were transferred into MSM medium containing 0.1% SE oil (v/v). In degradation studies, the following experimental groups were tested: immobilized cells and medium without any inoculum (negative control). Flask inoculated only with the BOBA1 in MSM medium was used as mycelium growth control. The samples were incubated at 25 ± 2 °C in a rotary incubator at 150 rpm. The samples were analysed at every 7th day during 28 days of degradation. BOD_5_ was calculated for the control and treated samples by Winkler titration method. The oil in the culture flasks and the adsorbed oil on the immobilized cells were extracted three times with n-hexane and dichloromethane (1:1). The yield during extraction process improves consistently with increase in solvent ratios till a point at which they stabilize. The extracts from each replicate were dried to a constant weight to determine the residual oil content. The rate of oil removal was quantified based on the amount of residual oil present in the samples. The residual organic phase was dehydrated in a column with 2 g of Na_2_SO_4_ and filtered through a 0.2 nylon membrane filter. The extracts were concentrated by rotary evaporation (Rotavapor model R110; Buchi Labortechnik, Switzerland) at ambient temperature and was redissolved in n-hexane.

### Effect of phosphate and nitrogen source

Effect of fertilizers on microbial degradation of oil was studied in MSM with 2% (v/v) immobilized cells and 0.1% (v/v) SE oil supplemented with 0.1% (w/v) of N and P sources, such as yeast extract, beef extract, NH_4_NO_3_, (NH_4_)_2_SO_4_, NH_4_Cl, (NH_4_)_2_SO_4,_CO(NH_2_)_2_, K_2_HPO_4_, KH_2_PO_4_ and NH_4_H_2_PO_4_. The culture flasks were incubated at 25 ± 2 °C under shaking conditions. Biodegradation experiment was carried out in duplicates up to 28 days and samples were analyzed every 7 days interval.

### Biodegradation studies at high pressure

High pressure reactors with the working volume of 5L were used to investigate the biodegradation under elevated pressures. The reactors were filled with MSM medium supplemented with 0.1% SE oil. The high pressure reactors were pressurized using nitrogen gas up to 10 MPa and incubated at 20 °C. These reactors can efficiently work under elevated pressure by simulating in situ deep-sea conditions without any deviation. The growth and multiplication of bacterial cells in high pressure were monitored by measuring total viable count and the total hydrocarbon concentration was analyzed to quantify the degree of biodegradation.

### Determination of oil biodegradation

The changes in total hydrocarbons (TH) of the SE oil samples were monitored over time by gravimetric method after extracting in 100 mL solvents (n-hexane, dichloromethane). The concentrated samples were subsequently analyzed by UV Spectrophotometer (UH 5300 UV/VIS, Hitachi High-Tech., Japan). FTIR studies were carried out for 7, 14, 21 and 28 days microbial treated samples in the range from 400 to 4000 cm^−1^, using SHIMADZU–IR Affinity-1 spectrophotometer, Japan. The Agilent technologies instrument (GC 7890 A, 240-MS/4000, USA) under external ionization mode using fused silica column HP-5 MS column (30 m × 0.32 mm × 0.25 µm) was used for GC–MS analysis of oil and degradation products. The ^1^H and ^13^C NMR experiments were carried out on 200 µL samples diluted in 400 µL of deuterated chloroform (99.5%) and analyzed at 40 °C on a high resolution liquid state NMR (Jeol ECA 500 MHz).

### Library preparation and genome sequencing

Genomic DNA was isolated from 10 mL of culture using QIAamp DNA mini kit (Qiagen, Valencia, USA) as per the protocol given by the manufacturer. The quality and purity of the genomic DNA was checked using agarose gel electrophoresis and Qubit 3 Fluorometer, respectively. Based on agarose gel electrophoresis, the quantity was verified using the Qubit dsDNA HS assay kit for precise measurements. The whole genome was sequenced by paired-end sequencing (150 × 2) with a Hiseq 4000 sequencing system (Illumina). The raw data obtained was processed with TrimGalore to remove the adapters and the low quality bases with Q-score less than 20 were filtered with FastQC. The pre-processed quality reads was used for the de novo genome assembly. SPAdes assembler (13.3.0) was used for de novo assembly after error-correction of sequenced reads. The assembled genome of BOBA1 was used for gene prediction using Augustus annotation and the predicted proteins similarity were searched against Uniprot fungi protein database using DIAMOND BLASTP program with an e-value of 10^–5^ for gene ontology and annotation. The value closer to zero illustrated the high score to the query sequence. Comparative genome analysis was performed with OrthoVenn and the metabolic pathways were determined.

## Conclusion

A novel hydrocarbonoclastic and piezotolerant deep-sea strain *A. sydowii* BOBA1 showed a high degradation capacity for PAHs and their complex mixture in SE oil hydrocarbon fractions within a retention period of 21 days. This strain degraded 71.2 and 82.5% of SE oil at 0.1 and 10 MPa high pressure culture conditions. Spectroscopic analyses and key functional annotations suggested that aromatic hydrocarbons were degraded into metabolites of the TCA cycle by phthalate, catechol and gentistate pathway. The complete genome analysis of BOBA1 consists of a 38.8 Mb single circular chromosome with 16,247 protein coding sequences. Specialized proteins and distinctive enzymes such as Rieske protein, siderophore, oxygenases, hydrolases, glutathione, arylsulfatase, peroxidase and dehydrogenase genes in the genome speculate the ability to survive in extremophilic environment and degrade toxic pollutants. These findings on hydrocarbon degradation at high pressure conditions and genome analysis provide insights into bioremediation related genes and their regulation to devise strategies for the remediation of hydrocarbon contaminated deep-sea environments.

## Supplementary Information

Supplementary Information 1.

Supplementary Information 2.

Supplementary Information 3.
