# Supplementary material for: Genome sequence analysis of deep sea Aspergillus sydowii BOBA1 and effect of high pressure on biodegradation of spent engine oil
Source: Sci Rep. 2021 Apr 30;11:9347. doi: 10.1038/s41598-021-88525-9 (PMC8087790; doi:10.1038/s41598-021-88525-9)
Supplement: Supplementary file 1 — Supplementary Information 1. [file 41598_2021_88525_MOESM1_ESM.docx]

Supplementary data

Data E1: Potential functions of unique genes of *A. sydowii* BOBA1
