# Supplementary material for: Genome sequence analysis of deep sea Aspergillus sydowii BOBA1 and effect of high pressure on biodegradation of spent engine oil
Source: Sci Rep. 2021 Apr 30;11:9347. doi: 10.1038/s41598-021-88525-9 (PMC8087790; doi:10.1038/s41598-021-88525-9)

**Genome Sequence Analysis of Deep Sea *Aspergillus sydowii* BOBA1 and Effect of High Pressure on Biodegradation of Spent Engine Oil**

A. Ganesh Kumar*, D. Manisha, K. Sujitha, D. Magesh Peter, R. Kirubagaran

and G. Dharani

***Marine Biotechnology Division,***

***National Institute of Ocean Technology,***

***Ministry of Earth Sciences (MoES), Government of India,***

***Chennai-600100, Tamil Nadu, India***

Running title: Hydrocarbonoclastic piezotolerant deep-sea fungus and whole genome analysis

**Corresponding Author**

***Dr. A. Ganesh Kumar, Project Scientist II,**

**Marine Biotechnology Division,**

**National Institute of Ocean Technology,**

**Ministry of Earth Sciences, Government of India, Chennai-600100,**

**Tamil Nadu, India.**

**Tel: +91 44 66783418.**

**Fax: +91 44 66783430.**

**Table S1:** Monooxygenase genes in the genome of *A. sydowii* BOBA1.

| **Monooxygenase** | | | | | |
| --- | --- | --- | --- | --- | --- |
| **S.No.** | **Protein ID(Subject ID)** | **% Identity** | **Protein Names** | **Gene name** | **Gene Ontology IDs** |
| 105 | tr|A0A137P558|A0A137P558_CONC2 | 45.7 | Putative monooxygenase | CONCODRAFT_39744 | GO:0004497; GO:0071949 |
| 133 | tr|A0A0J1BE66|A0A0J1BE66_9TREE | 65.8 | Flavin binding monooxygenase | CC85DRAFT_324708 | GO:0004497 |
| 429 | tr|A0A2H3D841|A0A2H3D841_ARMGA | 49.9 | Putative flavo protein monooxygenase acting on aromatic compound | ARMGADRAFT_1168888 | GO:0004497; GO:0071949 |
| 601 | tr|A0A0A2W0U3|A0A0A2W0U3_BEABA | 45.3 | Putative monooxygenase yxeK | BBAD15_g634 | GO:0004497; GO:0009058; GO:0016705; GO:0030170 |
| 857 | tr|A0A3D8RMZ1|A0A3D8RMZ1_9HELO | 61.5 | Putative pyrimidine monooxygenase RutA | BP6252_06448 | GO:0004497; GO:0006212; GO:0016705 |
| 1486 | tr|A0A0A2VZ03|A0A0A2VZ03_BEABA | 67.1 | Alkanesulfonate monooxygenase | BBAD15_g2837 | GO:0005524; GO:0005886; GO:0008509; GO:0008726; GO:0016021; GO:0016887 |
| 1495 | tr|A0A2G8N457|A0A2G8N457_9PSED | 44.2 | Nitrilotriacetate monooxygenase | CQW31_05310 | GO:0004497; GO:0016705 |
| 1496 | tr|A0A2L0UDD9|A0A2L0UDD9_9MICC | 45.4 | Monooxygenase | CVO76_06135 | GO:0004497; GO:0016627; GO:0050660 |
| 1497 | tr|A0A0A2VZ03|A0A0A2VZ03_BEABA | 38.2 | Alkanesulfonate monooxygenase | BBAD15_g2837 | GO:0005524; GO:0005886; GO:0008509; GO:0008726; GO:0016021; GO:0016887 |
| 2089 | tr|A0A2G8N170|A0A2G8N170_9PSED | 49.2 | Monooxygenase | CQW31_12010 | GO:0004497; GO:0016627; GO:0050660 |
| 2108 | sp|B2TEK6|NMO_PARPJ | 57.1 | Nitronate monooxygenase (NMO) (EC 1.13.12.-) (Propionate 3-nitronate monooxygenase) (P3N monooxygenase) | Bphyt_4144 | GO:0000166; GO:0009636; GO:0018580 |
| 2248 | tr|A0A2G7FFK8|A0A2G7FFK8_9EURO | 60.7 | Cytochrome P450 monooxygenase | AARAC_007610 | GO:0004497; GO:0005506; GO:0016021; GO:0016705; GO:0020037; GO:0046854 |
| 4512 | tr|A0A1L9TE22|A0A1L9TE22_9EURO | 99.4 | Ubiquinone biosynthesis monooxygenase COQ6, mitochondrial (EC 1.14.13.-) | COQ6 ASPSYDRAFT_46712 | GO:0006744; GO:0016709; GO:0016712; GO:0031314 |

**Table S2:** Dioxygenase genes in the genome of *A. sydowii* BOBA1*.*

| **Dioxygenase** | | | | | |
| --- | --- | --- | --- | --- | --- |
| **S.No.** | **Protein ID(Subject ID)** | **% Identity** | **Protein Names** | **Gene name** | **Gene Ontology IDs** |
| 45 | tr|A0A1B3F884|A0A1B3F884_9GAMM | 62.6 | Quercetin 2,3-dioxygenase  *Serratia* sp. YD25 | ATE40_001500 | GO:0051213 |
| 239 | tr|A0A2G8MZL7|A0A2G8MZL7_9PSED | 41.9 | Dioxygenase  *Pseudomonas* sp. 382 | CQW31_13260 | GO:0006725; GO:0008198; GO:0008270; GO:0016701; GO:0051213 |
| 313 | tr|A0A0A2W555|A0A0A2W555_BEABA | 46.2 | Putative quercetin 2,3-dioxygenase  *Beauveria bassiana* D1-5 | BBAD15_g283 | GO:0051213 |
| 572 | tr|A0A2G8N151|A0A2G8N151_9PSED | 46.5 | 4-hydroxyphenylpyruvate dioxygenase  *Pseudomonas* sp. 382 | CQW31_11850 | GO:0051213 |
| 744 | tr|A0A1C7NH06|A0A1C7NH06_9FUNG | 34.9 | Gamma-butyrobetaine dioxygenase  *Choanephora cucurbitarum* | BBOX1 A0J61_03680 | GO:0051213 |
| 1239 | tr|A0A1L9TFC4|A0A1L9TFC4_9EURO | 99.7 | Fe2OG dioxygenase domain-  *Aspergillus sydowii* CBS 593.65 | ASPSYDRAFT_32084 | GO:0005506; GO:0016706; GO:0031418 |
| 1482 | tr|A0A1B3FHJ0|A0A1B3FHJ0_9GAMM | 42.9 | Taurine dioxygenase  *Serratia* sp. YD25 | ATE40_019215 | GO:0051213 |
| 1619 | tr|A0A1L9TL55|A0A1L9TL55_9EURO | 100 | Fe2OG dioxygenase domain-containing protein  *Aspergillus sydowii* CBS 593.65 | ASPSYDRAFT_930069 | GO:0016491 |
| 2003 | tr|A0A2G8N612|A0A2G8N612_9PSED | 58.4 | Quercetin 2,3-dioxygenase  *Pseudomonas* sp. 382 | CQW31_00690 | GO:0051213 |
| 2109 | tr|A0A0D2GAP9|A0A0D2GAP9_XYLBA | 44.9 | Unplaced genomic scaffold supercont1.5, whole genome shotgun sequence  *Cladophialophora bantiana* CBS 173.52 | Z519_04252 | GO:0008941; GO:0019825; GO:0020037; GO:0051409; GO:0071949 |
| 2406 | tr|A0A1L9T0X3|A0A1L9T0X3_9EURO | 99.7 | Fe2OG dioxygenase  *Aspergillus sydowii* CBS 593.65 | ASPSYDRAFT_46329 | GO:0016491 |
| 5140 | tr|A0A1L9T5G0|A0A1L9T5G0_9EURO | 98.3 | Intradiol_Dioxygenas domain-containing protein, *Aspergillus sydowii* CBS 593.65 | ASPSYDRAFT_49723 | GO:0000981; GO:0003677; GO:0005634; GO:0006351; GO:0008270 |
| 6400 | tr|A0A1L9T9V6|A0A1L9T9V6_9EURO | 98.8 | Fe2OG dioxygenase domain-containing protein,  *Aspergillus sydowii* CBS 593.65 | ASPSYDRAFT_157659 | GO:0016491; GO:0046872 |
| 6676 | tr|A0A1L9T9K8|A0A1L9T9K8_9EURO | 99.5 | Cysteine dioxygenase (EC 1.13.11.20)  *Aspergillus sydowii* CBS 593.65 | ASPSYDRAFT_48325 | GO:0005506; GO:0017172; GO:0042412 |
| 7058 | tr|A0A1L9TCN7|A0A1L9TCN7_9EURO | 99.1 | Fe2OG dioxygenase domain-containing protein, *Aspergillus sydowii* CBS 593.65 | ASPSYDRAFT_155887 | GO:0016491; GO:0046872 |
| 7096 | tr|A0A1L9TXZ5|A0A1L9TXZ5_9EURO | 99.5 | 4-hydroxyphenylpyruvate dioxygenase, *Aspergillus sydowii* CBS 593.65 | ASPSYDRAFT_26323 | GO:0003868; GO:0009072; GO:0046872 |
| 7670 | tr|A0A1L9TM26|A0A1L9TM26_9EURO | 98.4 | Fe2OG dioxygenase domain-containing protein, *Aspergillus sydowii* CBS 593.65 | ASPSYDRAFT_56008 | GO:0016491 |
| 7784 | tr|A0A1L9T298|A0A1L9T298_9EURO | 97.9 | Fe2OG dioxygenase domain-containing protein, *Aspergillus sydowii* CBS 593.65 | ASPSYDRAFT_162316 | GO:0016491; GO:0046872 |
| 8653 | tr|A0A1L9SZQ7|A0A1L9SZQ7_9EURO | 99.4 | 1,2-dihydroxy-3-keto-5-methylthiopentene dioxygenase (EC 1.13.11.54) (Acireductone dioxygenase (Fe(2+)-requiring)) (ARD) (Fe-ARD), *Aspergillus sydowii* CBS 593.65 | ADI1 ASPSYDRAFT_51795 | GO:0005506; GO:0005634; GO:0005737; GO:0010309; GO:0019509 |
| 8718 | tr|A0A1L9TUL7|A0A1L9TUL7_9EURO | 96.4 | Fe2OG dioxygenase domain-containing protein  *Aspergillus sydowii* CBS 593.65 | ASPSYDRAFT_86622 | GO:0016491; GO:0046872 |
| 8796 | tr|A0A1L9TES7|A0A1L9TES7_9EURO | 99.7 | Deoxyhypusine hydroxylase (DOHH) (EC 1.14.99.29) (Deoxyhypusine dioxygenase) (Deoxyhypusine monooxygenase), *Aspergillus sydowii* CBS 593.65 | LIA1 ASPSYDRAFT_69065 | GO:0005634; GO:0005737; GO:0008612; GO:0019135; GO:0046872 |
| 8939 | tr|A0A1L9TUX0|A0A1L9TUX0_9EURO | 100 | 3-hydroxyanthranilate 3,4-dioxygenase (EC 1.13.11.6) (3-hydroxyanthranilate oxygenase) (3-HAO) (3-hydroxyanthranilic acid dioxygenase) (HAD) (Biosynthesis of nicotinic acid protein 1), *Aspergillus sydowii* CBS 593.65 | BNA1 ASPSYDRAFT_55821 | GO:0000334; GO:0005737; GO:0006569; GO:0008198; GO:0019805; GO:0034354; GO:0043420 |
| 9374 | tr|A0A1L9T7N4|A0A1L9T7N4_9EURO | 100 | Fe2OG dioxygenase domain-containing protein, *Aspergillus sydowii* CBS 593.65 | ASPSYDRAFT_183539 | GO:0005506; GO:0009058; GO:0016491 |
| 10610 | tr|A0A1L9TNQ4|A0A1L9TNQ4_9EURO | 99.6 | Fe2OG dioxygenase domain-containing protein, *Aspergillus sydowii* CBS 593.65 | ASPSYDRAFT_87604 | GO:0005506; GO:0016705; GO:0031418 |
| 10791 | tr|A0A1L9T4Y9|A0A1L9T4Y9_9EURO | 99.5 | 3-hydroxyanthranilate 3,4-dioxygenase (EC 1.13.11.6) (3-hydroxyanthranilate oxygenase) (3-HAO) (3-hydroxyanthranilic acid dioxygenase) (HAD) (Biosynthesis of nicotinic acid protein 1), *Aspergillus sydowii* CBS 593.65 | BNA1 ASPSYDRAFT_185220 | GO:0000334; GO:0005737; GO:0006569; GO:0008198; GO:0019805; GO:0034354; GO:0043420 |
| 11476 | tr|A0A1L9TIH2|A0A1L9TIH2_9EURO | 99.6 | Fe2OG dioxygenase domain-containing protein, *Aspergillus sydowii* CBS 593.65 | ASPSYDRAFT_151831 | GO:0016491 |
| 11727 | tr|A0A1L9TSK7|A0A1L9TSK7_9EURO | 100 | Fe2OG dioxygenase domain-containing protein, *Aspergillus sydowii* CBS 593.65 | ASPSYDRAFT_129237 | GO:0016491; GO:0046872 |
| 12402 | tr|A0A1L9TR83|A0A1L9TR83_9EURO | 99.7 | Fe2OG dioxygenase domain-containing protein, *Aspergillus sydowii* CBS 593.65 | ASPSYDRAFT_40512 | GO:0016491; GO:0046872 |
| 12442 | tr|A0A3A2ZHU0|A0A3A2ZHU0_9EURO | 79 | Glyoxalase/Bleomycin resistance protein/Dioxygenase superfamily,  *Aspergillus sclerotialis* | PHISCL_04985 | GO:0051213 |
| 13005 | tr|A0A254U4Y3|A0A254U4Y3_ASPNG | 61.8 | Fe2OG dioxygenase domain-containing protein, *Aspergillus niger* | ATCC64974_73660 | GO:0016491; GO:0046872 |
| 13016 | tr|A0A1L9PNK5|A0A1L9PNK5_ASPVE | 98.7 | Fe2OG dioxygenase domain-containing protein, *Aspergillus versicolor* CBS 583.65 | ASPVEDRAFT_72837 | GO:0016491; GO:0046872 |
| 13085 | tr|A0A1L9TR48|A0A1L9TR48_9EURO | 96.4 | Fe2OG dioxygenase domain-containing protein, *Aspergillus sydowii* CBS 593.65 | ASPSYDRAFT_195020 | GO:0016491; GO:0046872 |
| 13849 | tr|A0A1L9SYA4|A0A1L9SYA4_9EURO | 62 | Fe2OG dioxygenase domain-containing protein, *Aspergillus sydowii* CBS 593.65 | ASPSYDRAFT_52297 | GO:0016491 |
| 14357 | tr|A0A395MEJ5|A0A395MEJ5_9HYPO | 61.5 | Ferredoxin, *Fusarium* sp. FIESC_12 | FIE12Z_9468 | GO:0009055; GO:0046872; GO:0051213; GO:0051537 |
| 14407 | tr|A0A2L0UFZ2|A0A2L0UFZ2_9MICC | 64.4 | Tryptophan 2,3-dioxygenase (TDO) (EC 1.13.11.11) (Tryptamin 2,3-dioxygenase) (Tryptophan oxygenase) (TO) (TRPO) (Tryptophan pyrrolase) (Tryptophanase), *Arthrobacter agilis* | kynA CVO76_11390 | GO:0004833; GO:0019441; GO:0020037; GO:0046872 |
| 15237 | tr|A0A2G8N151|A0A2G8N151_9PSED | 41.7 | 4-hydroxyphenylpyruvate dioxygenase,  *Pseudomonas* sp. 382 | CQW31_11850 | GO:0051213 |
| 15694 | tr|A0A2L0UFV9|A0A2L0UFV9_9MICC | 69.7 | 3,4-dihydroxyphenylacetate 2,3-dioxygenase, *Arthrobacter agilis* | hpaD CVO76_11215 | GO:0051213 |
| 15744 | tr|A0A2G8N4A2|A0A2G8N4A2_9PSED | 63.9 | 4-hydroxyphenylpyruvate dioxygenase  *Pseudomonas* sp. 382 | hppD CQW31_05235 | GO:0003868; GO:0009072; GO:0046872 |
| 16007 | tr|A0A2L0UIV6|A0A2L0UIV6_9MICC | 50 | 2-nitropropane dioxygenase  *Arthrobacter agilis* | CVO76_16880 | GO:0004152; GO:0006207; GO:0018580; GO:0051213 |

**Table S3:** Epoxide hydrolase genes in the genome of *A. sydowii* BOBA1

| **Epoxide hydrolase** | | | | | |
| --- | --- | --- | --- | --- | --- |
| **S.No.** | **Protein ID(Subject ID)** | **% Identity** | **Protein Names** | **Gene name** | **Gene Ontology IDs** |
| 3385 | tr|A0A1L9TDK3|A0A1L9TDK3_9EURO | 99.2 | Leukotriene A(4) hydrolase (LTA-4 hydrolase) (EC 3.3.2.10) (EC 3.4.11.-), *Aspergillus sydowii* CBS 593.65 | ASPSYDRAFT_89166 | GO:0004301; GO:0005737; GO:0008237; GO:0008270 |

**Table S4:** Glutathione genes in the genome of *A. sydowii* BOBA1.

| **Glutathione** | | | | | |
| --- | --- | --- | --- | --- | --- |
| **S.No.** | **Protein ID(Subject ID)** | **% Identity** | **Protein Names** | **Gene name** | **Gene Ontology IDs** |
| 11 | tr|A0A0L0HKM3|A0A0L0HKM3_SPIPN | 49 | Glutathione peroxidase, *Spizellomyces punctatus* (strain DAOM BR117) | SPPG_02525 | GO:0004602; GO:0006979 |
| 152 | tr|A0A2G8MSB0|A0A2G8MSB0_9PSED | 58.1 | Glutathione S-transferase, *Pseudomonas* sp. 382 | CQW31_27205 | GO:0016740 |
| 342 | tr|A0A1B3F7N0|A0A1B3F7N0_9GAMM | 44.3 | Glutathione-regulated potassium-efflux system protein KefC (K(+)/H(+) antiporter), *Serratia* sp. YD25 | kefC ATE40_000140 | GO:0005887; GO:0015299; GO:0015503; GO:0015643; GO:0019899; GO:0051595 |
| 493 | tr|A0A2G8N353|A0A2G8N353_9PSED | 51.7 | Hydroxyacylglutathione hydrolase (EC 3.1.2.6) (Glyoxalase II) (Glx II), *Pseudomonas* sp. 382 | gloB CQW31_06405 | GO:0004416; GO:0019243; GO:0046872 |
| 520 | tr|A0A2G8N177|A0A2G8N177_9PSED | 58 | Glutathione reductase (GRase) (EC 1.8.1.7), *Pseudomonas* sp. 382 | CQW31_10955 | GO:0004362; GO:0005623; GO:0006749; GO:0009055; GO:0045454; GO:0050660; GO:0050661 |
| 609 | tr|A0A2G8N111|A0A2G8N111_9PSED | 58.3 | Lactoylglutathione lyase (EC 4.4.1.5) (Glyoxalase I) *Pseudomonas* sp. 382 | gloA CQW31_11685 | Pseudomonas sp. 382 |
| 650 | tr|A0A0J0XLV5|A0A0J0XLV5_9TREE | 47.2 | Gamma-glutamylcyclotransferase (EC 4.3.2.-) *Cutaneotrichosporon oleaginosum* | CC85DRAFT_285854 | GO:0003839; GO:0006751; GO:0061928 |
| 764 | tr|A0A365NG59|A0A365NG59_GIBIN | 62.4 | Glutathione S-transferase, Gibberella intermedia (Bulb rot disease fungus) *Fusarium proliferatum* | FPRO05_09097 | GO:0016740 |
| 844 | tr|A0A1B3FH76|A0A1B3FH76_9GAMM | 56.8 | Glutathione S-transferase, (pyrene degradation) *Serratia* sp. YD25 | ATE40_018515 | GO:0016740 |
| 897 | tr|A0A0A2VZT0|A0A0A2VZT0_BEABA | 54.6 | Gamma-glutamyltranspeptidase *Beauveria bassiana* D1-5 | BBAD15_g818 | GO:0006751; GO:0036374 |
| 1178 | tr|A0A2G8MTL9|A0A2G8MTL9_9PSED | 61.4 | Glutathione synthetase (EC 6.3.2.3) (GSH synthetase) (GSH-S) (GSHase) (Glutathione synthase) *Pseudomonas* sp. 382 | gshB CQW31_24790 | GO:0004363; GO:0005524; GO:0046872 |
| 1480 | tr|A0A1B3F9T0|A0A1B3F9T0_9GAMM | 75.1 | S-(hydroxymethyl)glutathione dehydrogenase (EC 1.1.1.284) *Serratia sp.* YD25 | ATE40_004480 | GO:0006069; GO:0008270; GO:0051903 |
| 1481 | tr|A0A2G8MZ12|A0A2G8MZ12_9PSED | 62.4 | S-formylglutathione hydrolase (EC 3.1.2.12) *Pseudomonas* sp. 382 | fghA CQW31_15605 | GO:0018738; GO:0046294; GO:0052689 |
| 2309 | tr|A0A2L0UG41|A0A2L0UG41_9MICC | 67.9 | Formaldehyde dehydrogenase, glutathione-independent *Arthrobacter agilis* | fdhA CVO76_11480 | GO:0008270; GO:0016491 |
| 2321 | tr|A0A2G8MSG1|A0A2G8MSG1_9PSED | 60 | Glutathione S-transferase family protein, *Pseudomonas* sp. 382 | CQW31_27140 | GO:0004364 |
| 2404 | tr|A0A1L9TFU7|A0A1L9TFU7_9EURO | 100 | GFA domain-containing protein, *Aspergillus sydowii* CBS 593.65 | ASPSYDRAFT_32352 | GO:0008270; GO:0046294; GO:0051907 |
| 3368 | tr|A0A317VBR9|A0A317VBR9_9EURO | 66.6 | Haloacid dehalogenase, *Aspergillus heteromorphus* CBS 117.55 | BO70DRAFT_373803 | GO:0004364; GO:0019120 |
| 3733 | tr|A0A1L9T704|A0A1L9T704_9EURO | 99.7 | S-formylglutathione hydrolase (EC 3.1.2.12), *Aspergillus sydowii* CBS 593.65 | ASPSYDRAFT_159636 | GO:0005737; GO:0018738; GO:0046294; GO:0052689 |
| 5938 | tr|A0A0A2W683|A0A0A2W683_BEABA | 42.3 | Glutathione transport system permease protein gsiC *Beauveria bassiana* D1-5 | BBAD15_g96 | GO:0016021; GO:0055085 |
| 6124 | tr|A0A1L9TWM1|A0A1L9TWM1_9EURO | 99.8 | Glutathione reductase (EC 1.8.1.7) *Aspergillus sydowii* CBS 593.65 | ASPSYDRAFT_141413 | GO:0004362; GO:0005737; GO:0006749; GO:0009055; GO:0045454; GO:0050660; GO:0050661 |
| 6699 | tr|A0A1L9TB25|A0A1L9TB25_9EURO | 99.6 | VOC domain-containing protein, lactoylglutathione lyase activity [GO:0004462]; metal ion binding [GO:0046872], *Aspergillus sydowii* CBS 593.65 | ASPSYDRAFT_47800 | GO:0004462; GO:0046872 |
| 7196 | tr|A0A1L9T8H1|A0A1L9T8H1_9EURO | 99.4 | GST N-terminal domain-containing protein *Aspergillus sydowii* CBS 593.65 | ASPSYDRAFT_48792 | GO:0004364 |
| 7994 | tr|A0A1L9TQ42|A0A1L9TQ42_9EURO | 89.7 | Glutathione S-transferase kappa (EC 2.5.1.18) *Aspergillus sydowii* CBS 593.65 | ASPSYDRAFT_43424 | GO:0004364; GO:0015035 |
| 8275 | tr|A1DLI3|A1DLI3_NEOFI | 38.3 | Glutathione-s-transferase, *Neosartorya* *fischeri* (strain ATCC 1020 / DSM 3700 / CBS 544.65 / FGSC A1164 / JCM 1740 / NRRL 181 / WB 181) (*Aspergillus* *fischerianus*) | NFIA_049950 | GO:0016740 |
| 8443 | tr|A0A1L9U062|A0A1L9U062_9EURO | 100 | Glutathione synthetase (GSH-S) (EC 6.3.2.3) *Aspergillus sydowii* CBS 593.65 | ASPSYDRAFT_39677 | GO:0000287; GO:0004363; GO:0005524; GO:0042803; GO:0043295 |
| 8574 | tr|A0A1L9TXW8|A0A1L9TXW8_9EURO | 99.8 | WD_REPEATS_REGION domain-containing protein, Aspergillus sydowii CBS 593.65 metallopeptidase activity [GO:0008237]; glutathione catabolic process [GO:0006751] | ASPSYDRAFT_84311 | GO:0006751; GO:0008237 |
| 9433 | tr|A0A1L9TQL7|A0A1L9TQL7_9EURO | 100 | Glutathione S-transferase kappa (EC 2.5.1.18) *Aspergillus sydowii* CBS 593.65 | ASPSYDRAFT_1166073 | GO:0004364; GO:0015035 |
| 9693 | tr|A0A1L9TK42|A0A1L9TK42_9EURO | 99.9 | Zn(2)-C6 fungal-type domain-containing protein *Aspergillus sydowii* CBS 593.65 | ASPSYDRAFT_30623 | GO:0000981; GO:0005634; GO:0006751; GO:0008270; GO:0036374 |
| 11472 | tr|A0A1L9TDY8|A0A1L9TDY8_9EURO | 100 | Lactoylglutathione lyase (EC 4.4.1.5) (Glyoxalase I) *Aspergillus sydowii* CBS 593.65 | ASPSYDRAFT_46804 | GO:0004462; GO:0046872 |
| 12125 | tr|A0A0L1JJL9|A0A0L1JJL9_ASPNO | 90.7 | S-formylglutathione hydrolase (EC 3.1.2.12) *Aspergillus nomius* NRRL 13137 | ANOM_000181 | GO:0005737; GO:0018738; GO:0046294; GO:0052689 |
| 12129 | tr|A0A1F7ZJF1|A0A1F7ZJF1_9EURO | 95.1 | S-(hydroxymethyl)glutathione dehydrogenase (EC 1.1.1.284) *Aspergillus bombycis* | ABOM_012074 | GO:0006069; GO:0008270; GO:0051903 |
| 12423 | tr|A0A1L9TF99|A0A1L9TF99_9EURO | 100 | S-(hydroxymethyl)glutathione dehydrogenase (EC 1.1.1.284) *Aspergillus sydowii* CBS 593.65 | ASPSYDRAFT_58643 | GO:0006069; GO:0008270; GO:0051903 |
| 12456 | tr|A0A1L9TDV8|A0A1L9TDV8_9EURO | 99.7 | S-(hydroxymethyl)glutathione dehydrogenase (EC 1.1.1 *Aspergillus sydowii* CBS 593.65.284) | ASPSYDRAFT_46765 | GO:0006069; GO:0008270; GO:0051903 |
| 12470 | tr|A0A1L9TKF5|A0A1L9TKF5_9EURO | 100 | Glutathione S-transferase kappa (EC 2.5.1.18) *Aspergillus sydowii* CBS 593.65 | ASPSYDRAFT_176175 | GO:0004364; GO:0015035 |
| 13586 | tr|A0A1L9TNF9|A0A1L9TNF9_9EURO | 100 | Glutathione peroxidase Aspergillus sydowii CBS 593.65 | ASPSYDRAFT_146286 | GO:0004602; GO:0006979 |
| 15983 | tr|H6UJ35|H6UJ35_CAJCA | 55.9 | Glutathione S-transferase *Cajanus cajan* **(**Pigeon pea) *(Cajanus indicus)* | NA | GO:0016740 |
| 14040 | tr|A0A2L0UE46|A0A2L0UE46_9MICC | 49.7 | Glutathione ABC transporter ATP-binding protein *Arthrobacter agilis* | CVO76_07470 | GO:0005524; GO:0015833; GO:0016887 |
| 14593 | tr|A0A1F7ZIJ5|A0A1F7ZIJ5_9EURO | 91.3 | Putative glutathione-dependent formaldehyde-activating enzyme (EC 4.4.1.22) (S-(hydroxymethyl)glutathione synthase) Aspergillus bombycis | ABOM_012080 | GO:0008270; GO:0046294; GO:0051907 |
| 14746 | tr|A0A0A2VIH3|A0A0A2VIH3_BEABA | 39.7 | Glutathione transport system permease protein gsiC Beauveria bassiana D1-5 | BBAD15_g8570 | GO:0016021; GO:0016787; GO:0055085 |
| 16182 | tr|A0A2L0UD61|A0A2L0UD61_9MICC | 82.4 | Glutathione-dependent reductase Arthrobacter agilis | CVO76_05620 | GO:0004364 |

**Table S5:** Arylsulfatase genes in the genome of *A. sydowii* BOBA1.

| **Arylsulfatase** | | | | | |
| --- | --- | --- | --- | --- | --- |
| **S.No** | **Protein ID(Subject ID)** | **% Identity** | **Protein Names** | **Gene name** | **Gene ontology IDs** |
| 1297 | tr|A0A1L9T5W7|A0A1L9T5W7_9EURO | 98.1 | Arylsulfatase (AS) (EC 3.1.6.1) (Aryl-sulfate sulphohydrolase) *(Aspergillus sydowii)* | ASPSYDRAFT_35505 | GO:0004065; GO:0018958 |
| 3280 | tr|A0A1L9T012|A0A1L9T012_9EURO | 97.7 | Arylsulfatase (AS) (EC 3.1.6.1) (Aryl-sulfate sulphohydrolase) *(Aspergillus sydowii)* | ASPSYDRAFT_213268 | GO:0004065; GO:0018958 |
| 10496 | tr|A0A1L9TFB2|A0A1L9TFB2_9EURO | 99.6 | Sulfatase domain-containing protein *(Aspergillus sydowii)* | ASPSYDRAFT_152782 | GO:0004065; GO:0018958 |

**Table S6:** Peroxidase genes in the genome of *A. sydowii* BOBA1.

| **Peroxidase** | | | | | |
| --- | --- | --- | --- | --- | --- |
| **S.No.** | **Protein ID(Subject ID)** | **% Identity** | **Protein Names** | **Gene name** | **Gene Ontology IDs** |
| 11 | tr|A0A0L0HKM3|A0A0L0HKM3_SPIPN | 46.4 | Glutathione peroxidase  *Spizellomyces punctatus*  *(strain DAOM BR117)* | SPPG_02525 | GO:0004602; GO:0006979 |
| 976 | tr|A0A2G8MW71|A0A2G8MW71_9PSED | 66.9 | OsmC family peroxiredoxin  *Pseudomonas* sp. 382 | CQW31_19930 | GO:0004601; GO:0006979 |
| 977 | tr|A0A2G8N725|A0A2G8N725_9PSED | 62.7 | Thiol peroxidase (Tpx) (EC 1.11.1.15) (Peroxiredoxin tpx) (Prx) (Thioredoxin peroxidase) *Pseudomonas* sp*.* 382 | tpx CQW31_02595 | GO:0005623; GO:0008379; GO:0045454 |
| 1972 | tr|A0A2G8N406|A0A2G8N406_9PSED | 50.7 | Catalase-related peroxidase (EC1.11.1.-)  *Pseudomonas* sp. 382 | CQW31_04980 | GO:0004096; GO:0006979; GO:0020037; GO:0046872 |
| 2463 | tr|A0A1L9TB14|A0A1L9TB14_9EURO | 100 | Peroxiredoxin (EC 1.11.1.15)  *Aspergillus* *sydowii* CBS 593.65 | ASPSYDRAFT_47837 | GO:0004601; GO:0005623; GO:0045454; GO:0051920 |
| 2838 | tr|A0A1L9PDU9|A0A1L9PDU9_ASPVE | 84.9 | Heme_Haloperoxidase domain-containing protein *Aspergillus* *versicolor* CBS 583.65 | ASPVEDRAFT_39082 | GO:0004601 |
| 4184 | tr|A0A1L9T5W1|A0A1L9T5W1_9EURO | 98.5 | Heme_Haloperoxidase domain-containing protein *Aspergillus sydowii* CBS 593.65 | ASPSYDRAFT_93666 | GO:0004601 |
| 5217 | tr|A0A1L9TJT8|A0A1L9TJT8_9EURO | 100 | Peroxidase (EC 1.11.1.)  *Aspergillus* *sydowii* CBS 593.65 | ASPSYDRAFT_44076 | GO:0004601; GO:0006979; GO:0020037; GO:0046872 |
| 6756 | tr|A0A1L9SZ07|A0A1L9SZ07_9EURO | 95.7 | Heme_Haloperoxidase domain-containing protein *Aspergillus sydowii* CBS 593.65 | ASPSYDRAFT_189715 | GO:0004601 |
| 6949 | tr|A0A1L9TU37|A0A1L9TU37_9EURO | 100 | Peroxidase (EC 1.11.1.)  *Aspergillus sydowii* CBS 593.65 | ASPSYDRAFT_144707 | GO:0004601; GO:0006979; GO:0020037; GO:0046872 |
| 6950 | tr|A0A1L9TU37|A0A1L9TU37_9EURO | 99.7 | Peroxidase (EC 1.11.1.)  *Aspergillus sydowii* CBS 593.65 | ASPSYDRAFT_144707 | GO:0004601; GO:0006979; GO:0020037; GO:0046872 |
| 8129 | tr|A0A1L9TBQ3|A0A1L9TBQ3_9EURO | 89.7 | Heme_Haloperoxidase domain-containing protein  *Aspergillus sydowii* CBS 593.65 | ASPSYDRAFT_33789 | GO:0004601 |
| 8472 | tr|A0A1L9T981|A0A1L9T981_9EURO | 100 | Peroxiredoxin (EC 1.11.1.15)  *Aspergillus sydowii* CBS 593.65 | ASPSYDRAFT_92197 | GO:0004601; GO:0005623; GO:0045454; GO:0051920 |
| 9317 | tr|A0A1L9T7X5|A0A1L9T7X5_9EURO | 99.8 | Heme_Haloperoxidase domain-containing protein *Aspergillus sydowii* CBS 593.65 | ASPSYDRAFT_183585 | GO:0004601 |
| 9452 | tr|A0A1L9U0X1|A0A1L9U0X1_9EURO | 100 | Catalase-peroxidase (CP) (EC 1.11.1.21) (Peroxidase/catalase) *Aspergillus sydowii* CBS 593.65 | katG ASPSYDRAFT_85307 | GO:0004096; GO:0006979; GO:0020037; GO:0042744; GO:0046872 |
| 10083 | tr|A0A1B3FDB9|A0A1B3FDB9_9GAMM | 56.2 | Deferrochelatase/peroxidase (EC1.11.1.-)  *Serratia* sp. YD25 | ATE40_011195 | GO:0004601; GO:0020037; GO:0033212; GO:0042597; GO:0046872 |
| 10265 | tr|A0A1L9T3Z9|A0A1L9T3Z9_9EURO | 94.8 | Heme_Haloperoxidase domain-containing protein *Aspergillus sydowii* CBS 593.65 | ASPSYDRAFT_50151 | GO:0004601 |
| 10833 | tr|A0A1L9TJX5|A0A1L9TJX5_9EURO | 100 | Peroxiredoxin (EC 1.11.1.15) *Aspergillus sydowii* CBS 593.65 | ASPSYDRAFT_149347 | GO:0004601; GO:0005623; GO:0045454; GO:0051920 |
| 13586 | tr|A0A1L9TNF9|A0A1L9TNF9_9EURO | 100 | Glutathione peroxidase  *Aspergillus sydowii* CBS 593.65 | ASPSYDRAFT_146286 | GO:0004602; GO:0006979 |
| 14113 | tr|A0A1X2G684|A0A1X2G684_9FUNG | 46.7 | Peroxiredoxin (EC 1.11.1.15)  *Hesseltinella vesiculosa* | DM01DRAFT_1328026 | GO:0004601; GO:0005623; GO:0045454; GO:0051920 |
| 14122 | tr|A0A372RAV2|A0A372RAV2_9GLOM | 51.9 | Peroxidase (EC 1.11.1.)  *Rhizophagus* sp. MUCL 43196 | C1646_694876 | GO:0004601; GO:0006979; GO:0020037; GO:0046872 |
| 14150 | tr|K2S857|K2S857_MACPH | 53.8 | Peroxidase (EC 1.11.1.)  *Macrophomina phaseolina* (MS6) | MPH_09855 | GO:0004601; GO:0006979; GO:0020037; GO:0046872 |
| 15515 | tr|A0A2T3Z8J3|A0A2T3Z8J3_9HYPO | 66.7 | Catalase-peroxidase (CP) (EC 1.11.1.21) (Peroxidase/catalase)  *Trichoderma asperellum* CBS 433.97 | katG M441DRAFT_140270 | GO:0004096; GO:0006979; GO:0020037; GO:0042744; GO:0046872 |
| 15902 | tr|A0A2L0UIS1|A0A2L0UIS1_9MICC | 64.5 | Thioredoxin-dependent thiol peroxidase  *Arthrobacter agilis* | CVO76_16865 | GO:0004601; GO:0005623; GO:0045454 |
| 16038 | tr|A0A2L0UH20|A0A2L0UH20_9MICC | 55.7 | Deferrochelatase/peroxidase (EC1.11.1.-)  *Arthrobacter agilis* | CVO76_13510 | GO:0004601; GO:0005623; GO:0020037; GO:0033212; GO:0046872 |
| 16174 | tr|A0A1B3FCK1|A0A1B3FCK1_9GAMM | 79.7 | Thiol peroxidase (Tpx)  (EC 1.11.1.15) (Peroxiredoxin tpx) (Prx) (Thioredoxin peroxidase)  *Serratia* sp. YD25 | tpx ATE40_009730 | GO:0005623; GO:0008379; GO:0045454 |

**Table S7:** Semialdehyde dehydrogenase genes in the genome of *A. sydowii* BOBA1.

| **Semialdehyde dehydrogenase** | | | | | |
| --- | --- | --- | --- | --- | --- |
| **S.No.** | **Protein ID(Subject ID)** | **% Identity** | **Protein Names** | **Gene name** | **Gene Ontology IDs** |
| 141 | tr|A0A1B3FHW9|A0A1B3FHW9_9GAMM | 62.7 | Methylmalonate-semialdehyde dehydrogenase (Acylating) *Serratia* sp. YD25 | ATE40_019695 | GO:0004491 |
| 154 | tr|A0A0L0H6X2|A0A0L0H6X2_SPIPN | 49.8 | Succinate-semialdehyde dehydrogenase (EC 1.2.1.16) *Spizellomyces punctatus* (strain DAOM BR117) | SPPG_08068 | GO:0004777; GO:0006540; GO:0009013; GO:0009450; GO:0034599 |
| 241 | tr|A0A2G8N5E8|A0A2G8N5E8_9PSED | 43.9 | Succinate-semialdehyde dehydrogenase I  *Pseudomonas* sp. 382 | CQW31_04320 | GO:0009013; GO:0009450 |
| 265 | tr|A0A2G8MZI2|A0A2G8MZI2_9PSED | 67 | Aspartate-semialdehyde dehydrogenase (ASA dehydrogenase) (ASADH) (EC 1.2.1.11) (Aspartate-beta-semialdehyde dehydrogenase) *Pseudomonas* sp. 382 | asd CQW31_14745 | GO:0004073; GO:0009088; GO:0009089; GO:0009097; GO:0019877; GO:0046983; GO:0050661; GO:0051287; GO:0071266 |
| 324 | tr|A0A1B3FGS9|A0A1B3FGS9_9GAMM | 55.1 | Succinate-semialdehyde dehydrogenase (NADP(+)) *Serratia* sp. YD25 | gabD ATE40_017890 | GO:0009013; GO:0009450 |
| 374 | tr|A0A2G8N642|A0A2G8N642_9PSED | 44.9 | N-acetyl-gamma-glutamyl-phosphate reductase (AGPR) (EC 1.2.1.38) (N-acetyl-glutamate semialdehyde dehydrogenase) (NAGSA dehydrogenase)  Pseudomonas sp. 382 | argC CQW31_00910 | GO:0003942; GO:0005737; GO:0006526; GO:0051287 |
| 390 | tr|A0A1B3FGD7|A0A1B3FGD7_9GAMM | 53.2 | Succinate-semialdehyde dehydrogenase (NADP(+)) *Serratia* sp. YD25 | gabD ATE40_016985 | GO:0009013; GO:0009450 |
| 722 | tr|A0A1B3FKC3|A0A1B3FKC3_9GAMM | 59.4 | N-succinylglutamate 5-semialdehyde dehydrogenase (EC 1.2.1.71) (Succinylglutamic semialdehyde dehydrogenase) (SGSD) *Serratia* sp. YD25 | astD ATE40_010910 | GO:0019544; GO:0019545; GO:0043824 |
| 942 | tr|A0A2G8N5E8|A0A2G8N5E8_9PSED | 61.8 | Succinate-semialdehyde dehydrogenase I *Pseudomonas* sp. 382 | CQW31_04320 | GO:0009013; GO:0009450 |
| 1022 | tr|A0A2G8N0R1|A0A2G8N0R1_9PSED | 65.9 | Gamma-glutamyl phosphate reductase (GPR) (EC 1.2.1.41) (Glutamate-5-semialdehyde dehydrogenase) (Glutamyl-gamma-semialdehyde dehydrogenase) (GSA dehydrogenase) *Pseudomonas* sp. 382 | proA CQW31_12750 | GO:0004350; GO:0005737; GO:0050661; GO:0055129 |
| 1755 | tr|A0A0F4YTH1|A0A0F4YTH1_TALEM | 70.6 | Malonate-semialdehyde dehydrogenase (Acetylating) (EC 1.2.1.18) *Rasamsonia emersonii* CBS 393.64 | T310_4576 | GO:0004491; GO:0018478 |
| 3265 | tr|A0A1L9T0B9|A0A1L9T0B9_9EURO | 96.2 | Aldedh domain-containing protein  GO:0004491~methylmalonate-semialdehyde dehydrogenase (acylating) activity *Aspergillus sydowii* CBS 593.65 | ASPSYDRAFT_163562 | GO:0004491 |
| 3270 | tr|A0A1L9T028|A0A1L9T028_9EURO | 96.7 | Aldedh domain-containing protein methylmalonate-semialdehyde dehydrogenase (acylating) activity [GO:0004491] *Aspergillus sydowii* CBS 593.65 | ASPSYDRAFT_163967 | GO:0004491 |
| 3788 | tr|A0A1L9T4R7|A0A1L9T4R7_9EURO | 97.1 | Multifunctional fusion protein [Includes: L-glutamate gamma-semialdehyde dehydrogenase (EC 1.2.1.88) (L-glutamate gamma-semialdehyde dehydrogenase); Delta-1-pyrroline-5-carboxylate dehydrogenase (P5C dehydrogenase)] *Aspergillus sydowii* CBS 593.65 | ASPSYDRAFT_159854 | GO:0003842; GO:0010133; GO:0016620 |
| 6074 | tr|A0A1L9TH00|A0A1L9TH00_9EURO | 99 | Multifunctional fusion protein [Includes: L-glutamate gamma-semialdehyde dehydrogenase (EC 1.2.1.88) (L-glutamate gamma-semialdehyde dehydrogenase); Delta-1-pyrroline-5-carboxylate dehydrogenase (P5C dehydrogenase)] *Aspergillus sydowii* CBS 593.65 | ASPSYDRAFT_202234 | GO:0003842; GO:0010133; GO:0016620 |
| 6454 | tr|A0A1L9TDP9|A0A1L9TDP9_9EURO | 100 | Aldedh domain-containing protein methylmalonate-semialdehyde dehydrogenase (acylating) activity [GO:0004491] *Aspergillus sydowii* CBS 593.65 | ASPSYDRAFT_90830 | GO:0004491 |
| 6466 | tr|A0A1L9TI37|A0A1L9TI37_9EURO | 97.7 | Multifunctional fusion protein [Includes: L-glutamate gamma-semialdehyde dehydrogenase (EC 1.2.1.88) (L-glutamate gamma-semialdehyde dehydrogenase); Delta-1-pyrroline-5-carboxylate dehydrogenase (P5C dehydrogenase)]  *Aspergillus sydowii* CBS 593.65 | ASPSYDRAFT_58277 | GO:0003842; GO:0010133; GO:0016620 |
| 10278 | tr|A0A397HFE0|A0A397HFE0_9EURO | 83.9 | Large subunit of alpha-aminoadipate reductase carbonate dehydratase activity [GO:0004089]; L-aminoadipate-semialdehyde dehydrogenase activity [GO:0004043]; phosphopantetheine binding [GO:0031177]; zinc ion *Aspergillus thermomutatus* | LYS2 CDV56_104955 | GO:0004043; GO:0004089; GO:0008270; GO:0009085; GO:0015976; GO:0031177 |
| 11939 | tr|A0A1L9TG40|A0A1L9TG40_9EURO | 100 | Aldedh domain-containing protein GO:0004350~glutamate-5-semialdehyde dehydrogenase activity;GO:0050661~NADP binding *Aspergillus sydowii* CBS 593.65 | ASPSYDRAFT_44719 | GO:0004350; GO:0006561; GO:0050661 |
| 11975 | tr|A0A1L9TXD8|A0A1L9TXD8_9EURO | 100 | Multifunctional fusion protein [Includes: L-glutamate gamma-semialdehyde dehydrogenase (EC 1.2.1.88) (L-glutamate gamma-semialdehyde dehydrogenase); Delta-1-pyrroline-5-carboxylate dehydrogenase (P5C dehydrogenase)] *Aspergillus sydowii* CBS 593.65 | ASPSYDRAFT_138758 | GO:0003842; GO:0010133; GO:0016620 |
| 12144 | tr|A0A1L9TKD6|A0A1L9TKD6_9EURO | 100 | Succinate-semialdehyde dehydrogenase (EC 1.2.1.16) *Aspergillus sydowii* CBS 593.65 | ASPSYDRAFT_30717 | GO:0004777; GO:0009013; GO:0009450 |
| 12200 | tr|A0A1U8QGT5|A0A1U8QGT5_EMENI | 75.2 | FAR1 domain-containing protein methylmalonate-semialdehyde dehydrogenase (acylating) activity [GO:0004491]  *Emericella nidulans* (strain FGSC A4 / ATCC 38163 / CBS 112.46 / NRRL 194 / M139) (*Aspergillus nidulans*) | AN0859.2 | GO:0004491 |
| 12369 | tr|A0A1L9TAU0|A0A1L9TAU0_9EURO | 98.5 | Aldedh domain-containing protein methylmalonate-semialdehyde dehydrogenase (acylating) activity [GO:0004491]  Aspergillus sydowii CBS 593.65 | ASPSYDRAFT_182266 | GO:0004491 |
| 13911 | tr|A0A1L9U0H0|A0A1L9U0H0_9EURO | 99.6 | Succinate-semialdehyde dehydrogenase (EC 1.2.1.16) *Aspergillus sydowii* CBS 593.65 | ASPSYDRAFT_75572 | GO:0004777; GO:0009013; GO:0009450 |
| 14049 | tr|A0A2L0UDK3|A0A2L0UDK3_9MICC | 53.9 | Gamma-glutamyl phosphate reductase (GPR) (EC 1.2.1.41) (Glutamate-5-semialdehyde dehydrogenase) (Glutamyl-gamma-semialdehyde dehydrogenase) (GSA dehydrogenase) *Arthrobacter agilis* | proA CVO76_06340 | GO:0004350; GO:0005737; GO:0050661; GO:0055129 |
| 14116 | tr|A0A0B7FU90|A0A0B7FU90_THACB | 38.2 | Succinate-semialdehyde dehydrogenase (EC 1.2.1.16) Thanatephorus cucumeris (strain AG1-IB / isolate 7/3/14) (Lettuce bottom rot fungus) (Rhizoctonia solani) | RSOLAG1IB_03675 | GO:0004777; GO:0009013; GO:0009450 |
| 14128 | tr|A0A1L9U0H0|A0A1L9U0H0_9EURO | 99.5 | Succinate-semialdehyde dehydrogenase (EC 1.2.1.16) *Aspergillus sydowii* CBS 593.65 | ASPSYDRAFT_75572 | GO:0004777; GO:0009013; GO:0009450 |
| 14552 | tr|A0A2L0UI31|A0A2L0UI31_9MICC | 61.1 | Methylmalonate-semialdehyde dehydrogenase (CoA acylating) *Arthrobacter agilis* | mmsA CVO76_15540 | GO:0004491 |
| 15093 | tr|A0A2L0UI31|A0A2L0UI31_9MICC | 50.5 | Methylmalonate-semialdehyde dehydrogenase (CoA acylating) Arthrobacter agilis | mmsA CVO76_15540 | GO:0004491 |
| 15182 | tr|A0A1L7WYY5|A0A1L7WYY5_9HELO | 50.5 | Related to Succinate-semialdehyde dehydrogenase [NADP(+)]*Phialocephala subalpina* | PAC_07864 | GO:0016620 |
| 15394 | tr|A0A0L0H6X2|A0A0L0H6X2_SPIPN | 45.8 | Succinate-semialdehyde dehydrogenase (EC 1.2.1.16) *Spizellomyces punctatus* (strain DAOM BR117) | SPPG_08068 | GO:0004777; GO:0006540; GO:0009013; GO:0009450; GO:0034599 |
| 15440 | tr|A0A167ESY4|A0A167ESY4_9ASCO | 43.8 | Succinate-semialdehyde dehydrogenase (EC 1.2.1.16) *Sugiyamaella lignohabitans* | UGA2 AWJ20_2004 | GO:0004777; GO:0009013; GO:0009450 |
| 15460 | tr|A0A2L0UI31|A0A2L0UI31_9MICC | 65.2 | Methylmalonate-semialdehyde dehydrogenase (CoA acylating) *Arthrobacter agilis* | mmsA CVO76_15540 | GO:0004491 |
| 15607 | tr|A0A2L0UJ19|A0A2L0UJ19_9MICC | 48.3 | N-acetyl-gamma-glutamyl-phosphate reductase (AGPR) (EC 1.2.1.38) (N-acetyl-glutamate semialdehyde dehydrogenase) (NAGSA dehydrogenase) *Arthrobacter agilis* | argC CVO76_04360 | GO:0003942; GO:0005737; GO:0006526; GO:0046983; GO:0051287 |

**Table S8:** Siderophore genes in the genome of *A. sydowii* BOBA1.

| **Siderophore** | | | | | |
| --- | --- | --- | --- | --- | --- |
| **S.No.** | **Protein ID(Subject ID)** | **% Identity** | **Protein Names** | **Gene name** | **Gene Ontology IDs** |
| 218 | tr|A0A1B3FDA8|A0A1B3FDA8_9GAMM | 32.8 | NADPH-dependent ferric siderophore reductase *Serratia* sp. YD25 | ATE40_011175 | GO:0016491 |
| 289 | tr|A0A2L0UD63|A0A2L0UD63_9MICC | 62.7 | Cobalamin/Fe(3+)-siderophore ABC transporter ATP-binding protein *Arthrobacter agilis* | CVO76_05605 | GO:0005524; GO:0016887 |
| 1222 | tr|A0A1B3F895|A0A1B3F895_9GAMM | 43.7 | TonB-dependent receptor  cell outer membrane [GO:0009279]; iron ion binding [GO:0005506]; signaling receptor activity [GO:0038023]; siderophore transport [GO:0015891] *Serratia* sp. YD25 | ATE40_001580 | GO:0005506; GO:0009279; GO:0015891; GO:0038023 |
| 1997 | tr|A0A2G8MU46|A0A2G8MU46_9PSED | 35.2 | NADPH-dependent ferric siderophore reductase  *Pseudomonas* sp. 382 | CQW31_24095 | GO:0016491 |
| 2099 | tr|A0A0A2VDP9|A0A0A2VDP9_BEABA | 39.5 | Ferrioxamine receptor  GO:0015891~siderophore transport  *Beauveria bassiana* D1-5 | BBAD15_g8708 | GO:0005506; GO:0015891; GO:0038023 |
| 2306 | tr|A0A0A2VV32|A0A0A2VV32_BEABA | 43.3 | Ferrichrome receptor FcuA  integral component of membrane [GO:0016021]; iron ion binding [GO:0005506]; signaling receptor activity [GO:0038023]; siderophore transport [GO:0015891]  *Beauveria bassiana* D1-5 | BBAD15_g2785 | GO:0005506; GO:0015891; GO:0016021; GO:0038023 |
| 4282 | tr|A0A2G8N4I7|A0A2G8N4I7_9PSED | 35.2 | TonB-dependent siderophore receptor  *Pseudomonas* sp. 382 | CQW31_05950 | GO:0009279; GO:0016021 |
| 11955 | tr|A0A3D8SKB1|A0A3D8SKB1_9EURO | 85.8 | Siderophore transporter, RhtX family  *Aspergillus mulundensis* | DSM5745_03253 | GO:0016021; GO:0055085 |
| 15703 | tr|A0A2L0UJ27|A0A2L0UJ27_9MICC | 50 | NADPH-dependent ferric siderophore reductase  *Arthrobacter agilis* | CVO76_05600 | GO:0016491 |

**Table S9:** Rieske domain-containing protein genes in the genome of *A. sydowii* BOBA1.

| **Rieske domain-containing protein** | | | | | |
| --- | --- | --- | --- | --- | --- |
| **S.No.** | **Protein ID(Subject ID)** | **% Identity** | **Protein Names** | **Gene name** | **Gene Ontology IDs** |
| 1325 | tr|A0A1L9T615|A0A1L9T615_9EURO | 98.3 | Rieske domain-containing protein. *Aspergillus sydowii* CBS 593.65 | ASPSYDRAFT_136086 | GO:0016021; GO:0016491; GO:0044237; GO:0051537 |
| 2307 | tr|A0A2G8N515|A0A2G8N515_9PSED | 66.5 | Rieske (2Fe-2S) protein. *Pseudomonas* sp. 382 | CQW31_03565 | GO:0005506; GO:0016491; GO:0044237; GO:0051537 |
| 3213 | tr|A0A1L9TWA9|A0A1L9TWA9_9EURO | 99.9 | Rieske domain-containing protein. *Aspergillus sydowii* CBS 593.65 | ASPSYDRAFT_38347 | GO:0008942; GO:0020037; GO:0051537 |
| 3636 | tr|A0A1L9TPK8|A0A1L9TPK8_9EURO | 95.6 | Rieske domain-containing protein. *Aspergillus sydowii* CBS 593.65 | ASPSYDRAFT_56702 | GO:0005506; GO:0016491; GO:0044237; GO:0051537 |
| 6523 | tr|A0A1L9TZH8|A0A1L9TZH8_9EURO | 100 | Cytochrome b-c1 complex subunit Rieske, mitochondrial (EC 7.1.1.8). *Aspergillus sydowii* CBS 593.65 | ASPSYDRAFT_139884 | GO:0005743; GO:0008121; GO:0051537; GO:0070469 |
| 6569 | tr|A0A1L9TC39|A0A1L9TC39_9EURO | 100 | Rieske domain-containing protein. *Aspergillus sydowii* CBS 593.65 | ASPSYDRAFT_91280 | GO:0016491; GO:0051537 |
| 7821 | tr|A0A1L9T438|A0A1L9T438_9EURO | 99.8 | Rieske domain-containing protein. *Aspergillus sydowii* CBS 593.65 | ASPSYDRAFT_93822 | GO:0016491; GO:0050660; GO:0051537 |
| 7995 | tr|A0A1L9TPY8|A0A1L9TPY8_9EURO | 100 | Rieske domain-containing protein. *Aspergillus sydowii* CBS 593.65 | ASPSYDRAFT_77625 | GO:0016491; GO:0051537 |
| 8207 | tr|A0A1L9SZS0|A0A1L9SZS0_9EURO | 90.4 | Rieske domain-containing protein. *Aspergillus sydowii* CBS 593.65 | ASPSYDRAFT_583532 | GO:0016491; GO:0044237; GO:0051537 |
| 8740 | tr|A0A1L9U0S0|A0A1L9U0S0_9EURO | 98.4 | Rieske domain-containing protein. *Aspergillus sydowii* CBS 593.65 | ASPSYDRAFT_138483 | GO:0016491; GO:0051537 |
| 11617 | tr|A0A1L9TQI9|A0A1L9TQI9_9EURO | 95.1 | Rieske domain-containing protein. *Aspergillus sydowii* CBS 593.65 | ASPSYDRAFT_128649 | GO:0005506; GO:0016491; GO:0044237; GO:0051537 |
| 13675 | tr|A0A1L9TQG9|A0A1L9TQG9_9EURO | 99.6 | Rieske domain-containing protein. *Aspergillus sydowii* CBS 593.65 | ASPSYDRAFT_75729 | GO:0005506; GO:0016491; GO:0044237; GO:0051537 |
| 13676 | tr|A0A1L9TQG9|A0A1L9TQG9_9EURO | 99 | Rieske domain-containing protein. *Aspergillus sydowii* CBS 593.65 | ASPSYDRAFT_75729 | GO:0005506; GO:0016491; GO:0044237; GO:0051537 |
| 15153 | tr|A0A067TU81|A0A067TU81_GALM3 | 33.7 | Rieske domain-containing protein. *Galerina marginata* (strain CBS 339.88) | GALMADRAFT_235573 | GO:0008942; GO:0020037; GO:0042128; GO:0050660; GO:0050661; GO:0051537 |
| 15257 | tr|A0A2H1GZS0|A0A2H1GZS0_ZYMTR | 45 | Rieske domain-containing protein. *Zymoseptoria tritici* ST99CH_1E4 | ZT1E4_G9794 | GO:0008942; GO:0020037; GO:0051537 |

**Table S10:** Unique genes facilitating tolerance to high pressure in the genome of *A. sydowii* BOBA1.

| **Genes facilitate tolerance to high pressure** | | | | | |
| --- | --- | --- | --- | --- | --- |
| **S.No.** | **Protein ID(Subject ID)** | **% Identity** | **Protein Names** | **Gene name** | **Gene Ontology IDs** |
| 925 | tr|A0A2L0UD11|A0A2L0UD11_9MICC | 83.6 | UvrABC system protein B (Protein UvrB) (Excinuclease ABC subunit B) | uvrB CVO76_05305 | GO:0003677; GO:0005524; GO:0005737; GO:0006289; GO:0009380; GO:0009381; GO:0009432; GO:0016887 |
| 1342 | tr|A0A2G8MZF7|A0A2G8MZF7_9PSED | 73.1 | UvrABC system protein B (Protein UvrB) (Excinuclease ABC subunit B) | uvrB CQW31_14640 | GO:0003677; GO:0005524; GO:0005737; GO:0006289; GO:0009380; GO:0009381; GO:0009432; GO:0016887 |
| 121 | tr|A0A2G8MWI0|A0A2G8MWI0_9PSED | 54.5 | Two-component system response regulator UvrY | CQW31_19720 | GO:0000160; GO:0003677; GO:0006355 |
| 122 | tr|A0A2G8MWR5|A0A2G8MWR5_9PSED | 57 | UvrABC system protein C (Protein UvrC) (Excinuclease ABC subunit C) | uvrC CQW31_19725 | GO:0003677; GO:0005737; GO:0006289; GO:0009380; GO:0009381; GO:0009432 |
| 452 | tr|A0A2N0QHW1|A0A2N0QHW1_9GLOM | 79.9 | UvrA_inter domain-containing protein (Fragment) | RhiirA1_485742 | - |
| 520 | tr|A0A0A2VTY0|A0A0A2VTY0_BEABA | 70 | UvrABC system protein A | BBAD15_g3536 | GO:0003677; GO:0005524; GO:0006289; GO:0009380; GO:0016887 |
| 556 | tr|A0A2G8N057|A0A2G8N057_9PSED | 57.1 | UvrABC system protein A (UvrA protein) (Excinuclease ABC subunit A) | uvrA CQW31_12320 | GO:0003677; GO:0005524; GO:0005737; GO:0006289; GO:0008270; GO:0009380; GO:0009381; GO:0009432; GO:0016887 |
| 739 | tr|A0A1B3FHA8|A0A1B3FHA8_9GAMM | 69.9 | UvrABC system protein A (UvrA protein) (Excinuclease ABC subunit A) | uvrA ATE40_018595 | GO:0003677; GO:0005524; GO:0005737; GO:0006289; GO:0008270; GO:0009380; GO:0009381; GO:0009432; GO:0016887 |
| 918 | tr|A0A2G8N057|A0A2G8N057_9PSED | 67.2 | UvrABC system protein A (UvrA protein) (Excinuclease ABC subunit A) | uvrA CQW31_12320 | GO:0003677; GO:0005524; GO:0005737; GO:0006289; GO:0008270; GO:0009380; GO:0009381; GO:0009432; GO:0016887 |
| 1096 | tr|A0A2G8N057|A0A2G8N057_9PSED | 74.9 | UvrABC system protein A (UvrA protein) (Excinuclease ABC subunit A) | uvrA CQW31_12320 | GO:0003677; GO:0005524; GO:0005737; GO:0006289; GO:0008270; GO:0009380; GO:0009381; GO:0009432; GO:0016887 |
| 1136 | tr|A0A1B3F995|A0A1B3F995_9GAMM | 70.9 | D-alanine--D-alanine ligase (EC 6.3.2.4) (D-Ala-D-Ala ligase) (D-alanylalanine synthetase) | ddl ATE40_003510 | GO:0005524; GO:0005737; GO:0008360; GO:0008716; GO:0009252; GO:0046872; GO:0071555 |
| 768 | tr|A0A2L0UJ85|A0A2L0UJ85_9MICC | 55.7 | D-alanine--D-alanine ligase (EC 6.3.2.4) (D-Ala-D-Ala ligase) (D-alanylalanine synthetase) | ddl CVO76_06815 | GO:0005524; GO:0005737; GO:0008360; GO:0008716; GO:0009252; GO:0046872; GO:0071555 |
| 637 | tr|A0A2L0UI23|A0A2L0UI23_9MICC | 55.8 | Alanine dehydrogenase (EC 1.4.1.1) | ald CVO76_15485 | GO:0000166; GO:0000286; GO:0042853 |
| 807 | tr|A0A2L0UI23|A0A2L0UI23_9MICC | 65.5 | Alanine dehydrogenase (EC 1.4.1.1) | ald CVO76_15485 | GO:0000166; GO:0000286; GO:0042853 |
| 1016 | tr|A0A2L0UI23|A0A2L0UI23_9MICC | 67.4 | Alanine dehydrogenase (EC 1.4.1.1) | ald CVO76_15485 | GO:0000166; GO:0000286; GO:0042853 |
| 1181 | tr|A0A2G8MWB7|A0A2G8MWB7_9PSED | 62.9 | Radical SAM protein | CQW31_19980 | GO:0003824; GO:0051536 |
| 164 | tr|A0A1B3FC94|A0A1B3FC94_9GAMM | 50.1 | Cytochrome C | ATE40_009120 | GO:0005506; GO:0009055; GO:0016020; GO:0016614; GO:0020037 |
| 924 | tr|A0A2L0UCR1|A0A2L0UCR1_9MICC | 84.6 | Cytochrome ubiquinol oxidase subunit I | CVO76_04840 | GO:0005886; GO:0009055; GO:0016021; GO:0019646; GO:0046872; GO:0070069 |
| 1318 | tr|A0A0A2VY18|A0A0A2VY18_BEABA | 64.6 | Cytochrome c oxidase subunit 1 (EC 1.9.3.1) | BBAD15_g3082 | GO:0004129; GO:0005743; GO:0006119; GO:0008827; GO:0015990; GO:0016021; GO:0019646; GO:0020037; GO:0046872; GO:0070469 |
| 613 | tr|A0A0A2W5D4|A0A0A2W5D4_BEABA | 62.8 | Glycerol-3-phosphate transporter | BBAD15_g145 | GO:0015169; GO:0016021 |
| 835 | tr|A0A2L0UAT5|A0A2L0UAT5_9MICC | 84.5 | Phosphate import ATP-binding protein PstB (EC 7.3.2.1) (ABC phosphate transporter) (Phosphate-transporting ATPase) | pstB CVO76_01015 | GO:0005315; GO:0005524; GO:0005886; GO:0015415; GO:0016887 |
| 944 | tr|A0A0A2W5D4|A0A0A2W5D4_BEABA | 87 | Glycerol-3-phosphate transporter | BBAD15_g145 | GO:0015169; GO:0016021 |
| 94 | tr|A0A1B3FET6|A0A1B3FET6_9GAMM | 32.4 | Alkyl hydroperoxide reductase | ATE40_013940 | GO:0005623; GO:0016491; GO:0045454 |
| 407 | tr|A0A0A2WL17|A0A0A2WL17_BEABA | 68.2 | Alkyl hydroperoxide reductase subunit C | BBAD15_g255 | GO:0005623; GO:0006979; GO:0045454; GO:0051920 |
| 729 | tr|A0A0A2WL17|A0A0A2WL17_BEABA | 77.4 | Alkyl hydroperoxide reductase subunit C | BBAD15_g255 | GO:0005623; GO:0006979; GO:0045454; GO:0051920 |

**Supplementary figure F1**: GCMS profile of *A. sydowii* BOBA1 cells (mycelium growth control)


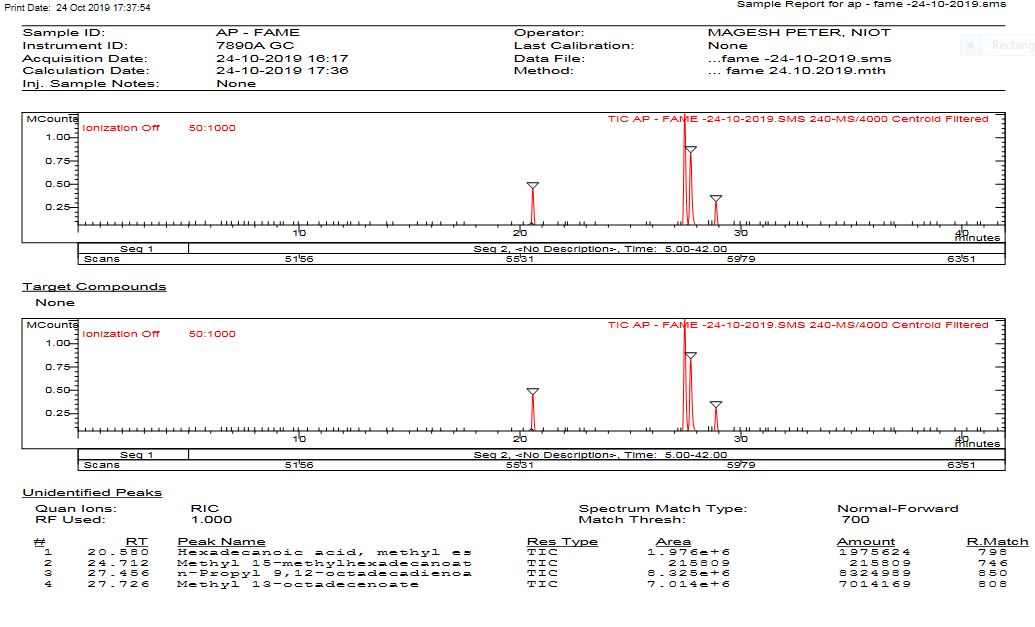


**Supplementary figure F2**: OrthoVenn results: Comparative genome analysis of *A. sydowii* BOBA1 with *A. sydowii* CBS 593.65.


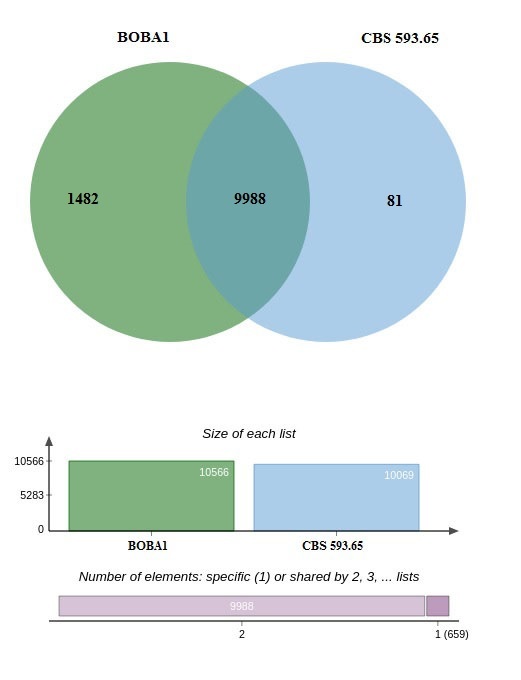

Supplement: Supplementary file 3 — Supplementary Information 3. [file 41598_2021_88525_MOESM3_ESM.doc]
